# Developing a strategic understanding of telehealth service adoption for COPD care management: A causal loop analysis of healthcare professionals

**DOI:** 10.1371/journal.pone.0229619

**Published:** 2020-03-05

**Authors:** Violeta Gaveikaite, Casandra Grundstrom, Katerina Lourida, Stefan Winter, Rita Priori, Ioanna Chouvarda, Nicos Maglaveras

**Affiliations:** 1 Laboratory of Computing, Medical Informatics and Biomedical Imaging Technologies, Aristotle University of Thessaloniki, Thessaloniki, Greece; 2 Department of Chronic Disease Management, Philips Research, Eindhoven, The Netherlands; 3 M3S, Faculty of Information Technology and Electrical Engineering, University of Oulu, Oulu, Finland; 4 Department of Chronic Disease Management, Philips Research, Aachen, Germany; 5 Department of IEMS, McCormick School of Engineering, Northwestern University, Evanston, Illinois, United States of America; Universite de Bretagne Occidentale, FRANCE

## Abstract

**Background:**

Telehealth services can improve the quality of health services for chronic obstructive pulmonary disease (COPD) management, but the clinical benefits for patients yet not clear. It is crucial to develop a strategy that supports the engagement of healthcare professionals to promote the sustainable adoption of telehealth services further. The aim of the study was to show how variables related to the perception of telehealth services for COPD by different healthcare professionals interact to influence its adoption and to generate advice for future telehealth service implementation.

**Methods:**

Data was thematically synthesized from published qualitative studies to create causal loop diagrams, further validated by expert interviews. These diagrams visualize dependencies and their polarity between different variables.

**Results:**

Adoption of telehealth services from the nurse’s perspective is directly affected by change management and autonomous decision making. From the physician’s perspective, perceived value is the most important variable. Physical activity management and positive user experience are considered affecting perceived value for physiotherapists. There is no consensus where self-management services should be positioned in the COPD care pathway.

**Conclusion:**

Our results indicate how complex interactions between multiple variables influence the adoption of telehealth services. Consequently, there is a need for multidimensional interventions to achieve adoption. Moreover, key variables were identified that require attention to ensure success of telehealth services. Furthermore, it is necessary to explore where self-management services are best positioned in the care pathway of COPD patients.

## Introduction

Chronic obstructive pulmonary disease (COPD) is a chronic inflammatory lung disease of which the prevalence continues to rise worldwide [1]. This implies an increasing demand for healthcare services [2,3], especially for the management of the highly comorbid [4] and elderly [5] COPD patients. Early recognition of COPD exacerbation or self-management education are some of the examples for services, which are given too little attention in healthcare organizations providing care to COPD patients [6]. In the current crisis of healthcare organizations, an aging population and lack of healthcare professionals (HCPs) for example, the demand for such services cannot be fully met [7,8]. To address this demand, telehealth services are explored [6] as they promise to provide timely care with lower associated costs [9]. Telehealth(TH) is defined as the ongoing and remote exchange of data between patients at home and HCPs as part of disease management [10]. Despite the many beneficial patient and organizational outcomes reported in COPD care management, [11] TH is currently not being implemented at a large scale in routine clinical care [12].

Implementation of new healthcare services into routine clinical care, including its obstacles, has received a lot of attention in recent years [13,14]. Considering TH services for COPD care management, many variables influencing the adoption were reported in randomized clinical trials (RCT) [15,16]. However, little is known about their complex interactions and influence on each other [17]. Brunton et al. [17] identified three themes influencing user experiences related to the TH services for COPD care management: influence on moral dilemmas of seeking help; transforming interactions between users and reconfiguration of ways of work. Another review focused on the acceptance by frontline staff of TH for COPD or mixed with chronic heart failure patients [18]. In addition to variables associated with implementation, the “pure” RCT design is criticized as not capturing other contextual variables [19]. Therefore, there is a need for RCTs to be extended to different designs falling under the umbrella term “complex interventions” [20].

The perceptions of HCPs towards the implementation of new services are very important [17,21]. Despite this importance, extant research is underdeveloped and limited evidence has elucidated the role HCPs play in the effectiveness of TH [22]. However, it is well known that a negative perception of HCPs influences the success or failure during implementation of new services, such as TH [21]. There is some evidence available that quantifies the influence of specific variables on the adoption of TH from the perspective of professionals. For example, adoption of TH influences the relationship between HCPs [23,24]. In order to engage HCPs in the process of adoption of TH for COPD, it is crucial to identify HCPs-specific variables and their dependencies.

There is an ongoing discussion in peer-reviewed literature regarding how research should progress in the field of COPD management supported by TH. One strategy may be to temporarily suspend TH research for COPD care management [25] whilst an alternative strategy is to explore the underlying mechanisms which hinder successful implementation [19]. A system dynamics approach permits the exploration of underlying mechanisms and helps to create scenarios which facilitate change management in the organization [26,27]. In the field of TH for COPD care management, there is a lack of publications that address how certain variables influence HCPs in the adoption of TH. This insufficiency complicates the potential transformation of qualitative knowledge into the solid conceptualization of the system [26]. Findings from such a study would support stakeholders that are involved in the actual TH implementation to reach a consensus, improve engagement in the adoption of TH tools, as well as generate innovative ideas and identify data gaps [26,28]. Thus, system dynamics would serve as a communication tool for informed policy decision making and have implications for future research.

We hypothesize that knowing, which variables affect TH adoption, is insufficient. Indicating a clear need to step back to explore the mechanisms occurring during the adoption of TH. Moreover, there is a need to understand the perception of different HCPs towards these variables, their dynamics and its influence on the adoption of TH for COPD. The aim of this study is to develop three causal loop diagrams (CLDs) representing the adoption process for TH services in COPD care management for each of the three stakeholders. Moreover, the aim is to generate advice for an adoption strategy to be used in future research or policy projects for TH in COPD.

## Methods

### Step 1: Data and sample

The research was guided by the methodological approach from Yourkavitch et al. [29] and Flax et al. [30]. Data for the CLDs was initially collected through a literature search for available articles on HCPs’ perspectives on TH for COPD management. Two co-authors (VG and KL) conducted a systematic literature search in PubMed and Embase in July of 2018 (S1 Appendix). The screening inclusion criteria were: not mixed COPD patient’s population (e.g. without asthma cases), clearly defined HCP role or title (e.g. secondary care practitioner), qualitative study design, English language, peer reviewed paper (including conference proceedings), TH intervention and study performed in Europe. Two co-authors (VG and CG) performed full-text reading based on criteria defined above. Relevant articles were included in the final synthesis. Additional articles were chosen by a literature snowballing technique [31] and confirmed for inclusion by co-authors (VG and CG).

Included articles were prepared for data extraction and are presented as part of the results in this study. A qualitative evidence synthesis (QES) approach was employed to harmonize HCPs’ perceptions and attitudes in the adoption of TH services [32][33][34]. A thematic synthesis was chosen as a suitable approach to interpret stratified constructs of TH adoption from the included QES literature [34]. Further, the thematic synthesis is a straightforward yet powerful approach for informing policymakers and HCPs regarding relevant insights into health technology (such as TH); complementary to the research aim of disseminating actionable findings [33][34]. As not all included articles were rich in qualitative data, the flexibility of the thematic synthesis allows interpretation of thin qualitative data as usable for developing aggregate yet descriptive themes [33][35].

Excerpts of empirical evidence from the included articles were extracted in the form of quotes from interviews or observations from ethnography. Before the coding process could take place, extracted excerpts were familiarized and grouped into either a barrier or facilitator of TH by determining whether the narrative described a limiting or enabling effect of TH. Excerpts were then further classified using colors to denote an HCP category: nurse, physiotherapist or physician. To begin the thematic synthesis, co-authors (VG and CG) independently coded each excerpt, no predetermined categories were used. A code is a label assigned to raw data to shortly describe excerpts [36]. Then, the two authors agreed on cohesive code names and synthesized codes to create granular descriptive themes consisting of variables that limit or enable TH adoption (cause or effect variables). Analytical themes were also developed from the descriptive themes; however, it was determined that the analytical themes lacked the granularity required for CLDs and are not discussed in this paper [35]. Findings from the QES were transformed to intuitive variables in each of the three HCP categories to develop relevant feedback loops in the subsequent stage.

### Step 2: Causal loop diagramming

To determine dependencies between variables that affect TH adoption for COPD care management, causal loop diagrams (CLDs) were used. Each of the three HCP’s perspectives were included: nurses, physicians and physiotherapists. To develop a CLD, the terms (Table 1) proposed by system dynamics publications were used [37,38].

**Table 1:** CLD elements and notations.

	Cause variable	Effect variable	Delay	Polarity	Dependency
**Graphical representation**	A	B	//	Positive polarity: ‘+’ Dotted line in the figure and negative polarity: ‘-‘	→ (long arrow)
**Definition**	Variable which causes effect in another variable	Variable which is affected by the cause variable	Arrow with 2 short lines across the causal link shows that the causal link appears with a delay in time	Positive polarity shows a positive relationship between 2 variables: if A increases, then B increases and if A decreases, then B decreases);Negative polarity shows an opposite relationship between the two variables: if A in, B decreases and as A decreases, B increases).	The cause-effect relationship between two variables. The thickness of the arrow exemplifies the published variable frequency in the relationships. Dashed arrows show probable relationships
**Example**	“When the knowledge gap diminishes the value perceived by physiotherapists increases”				
**Example translated into CLD elements**	“Knowledge Gap” *	“Value perceived”	With delay	Negative	“Knowledge gap” -> “Value perceived”

* Words in the brackets correspond to the variables used in the causal loop diagrams.

In the result section, words in the brackets correspond to the variables used in the CLD. In our diagrams feedback loops, which look like a closed circle and depict a sequence of dependencies which start and end with the same variable, were identified. These loops may be reinforcing or balancing (S3 Table). In reinforcing loops, variables influence each other in the same direction while in balancing loops they influence each other in opposite ways. In system dynamics research, analysis of feedback loops in the system is key to identify healthcare cycles and reinforce or attenuate them by appropriate policies [26]. To create a graphical representation of the loops, Vensim software (Ventana Systems, Harvard, MA, version PLE x32) was used. To clarify the variables, dependencies and polarities, the CLDs were reiterated by matching the context table to the most recent iteration of the CLD. Changes in the CLDs were documented. Variables not related to the main outcome were removed in the results section. All primary variables can be found in the initial drafts (S1 Table). Considering our aim to provide evidence to relevant stakeholders on which variables are considered by the different HCPs to be crucial, it was explored how often each of the variables are exposed to or are exposing other variables. Narrative comments were provided to those variables that require special attention by the policymakers makers. Specific feedback loops were identified as key areas of the data and are displayed in a separate table.

### Step 3: Diagram validation

The expert consultants were recruited from the authors of the included QES articles based on their role: 2 nurses, 2 physicians and 2 physiotherapists. Experts were required to confirm their extensive work in the COPD field (at least three years) and use of TH services for COPD care management. Initially, no nurses accepted the invitation to participate. Instead, champions in the field of COPD working with TH were contacted to offer support with recruitment of nurses for validation. Following work of previously published CLDs’ validation procedures [28][39], a target of two HCPs for each category was determined to be suitable. The validation procedure was performed by the primary author (VG) through a guided teleconference interview. The interviews lasted on average 60 minutes and followed an amended variables validation process regarding variable clarity, completeness, polarities, dependencies, and delays [40]. During the validation interviews, the initial synthesized findings were presented and discussed with the consultants regarding their accuracy (S2 Table). Validation evidence was incorporated in the CLDs based on the outcomes of the validation process (S1 Fig).

## Results

### Step 1: Data and sample

256 articles were screened which were found via a database search and 30 from snowballing. After removing duplicates and second stage full article reading, 17 articles published from 2004 to 2019 were included in the QES (Table 2). The qualitative studies were mostly performed in Denmark and England. Nurses were the predominant research participants, making up 15 of the 17 included articles. Whereas physicians and physiotherapists make up 8 and 4 of the articles respectively.

**Table 2:** List of articles used for data extraction and qualitative evidence synthesis.

Year	Authors	Country	HCPs Job Position Titles	Total Number of HCPs in Each Category		
				*Nurse*	*Physio therapist*	*Physicians*
**2019**	Nickelsen[41]	Denmark	Nurses, Doctors	10		4
**2017**	Nickelsen[24]	Denmark	Nurses, Doctors	10		4
**2015**	Rosenbek-Minet[42]	Denmark	Physiotherapists		2	
**2013**	Dinesen[43]	Denmark	Nurses at the hospital, Nurses at the healthcare center, District Nurses, Doctors at the hospital, General Practitioners	18		8
**2018**	Orme[44]	England	COPD Specialist Nurses, Ward nurses, Physiotherapists, Doctors	17	6	2
**2016**	Fitzsimmons [45]	England	Nurses	3		
**2014**	MacNeill [46]	England	Telehealth monitoring nurses, Community matrons, General Practitioners	23		9
**2014**	Odeh [47]	England	Practice nurses	7		
**2008**	Mair [16]	England	Specialist respiratory nurses	11		
**2004**	Hibbert [48]	England	Nurses	12		
**2017**	Segato [49]	Italy	Nurses, Physicians, General Practitioners	3*		4*
**2017**	Vorrink [50]	Netherlands	Physiotherapists		24	
**2017**	Barken [51]	Norway	Nurses	3		
**2013**	Fairbrother [15]	Scotland	Primary care nurses, Secondary care nurses, Research nurses, Community respiratory physiotherapists, General Practitioners	2*	2*	
**2012**	Fairbrother[52]	Scotland	Primary care nurses, Secondary care nurses, Research nurses, Telemonitoring physiotherapists, General Practitioners	1*	3*	2*
**2012**	Roberts [53]	Scotland	Community nurses, Specialist practice nurse, Respiratory nurse specialist, General Practitioners	5		1
**2012**	Ure [54]	Scotland	Practice nurses, Hospital-based respiratory nurses, community nurse managers, physiotherapists, General Practitioners	12	3	4

*Where exact number of participants were not written, authors included the least number of participants described in excerpts

### Step 2–3: Causal loop diagramming and validation

Our main outcome was the adoption of TH for COPD care management. The adoption of TH is described as a result of a lengthy decision-making process, which in later stage ensures the sustainability into the current healthcare setting practices [55].

**Fig 1:**
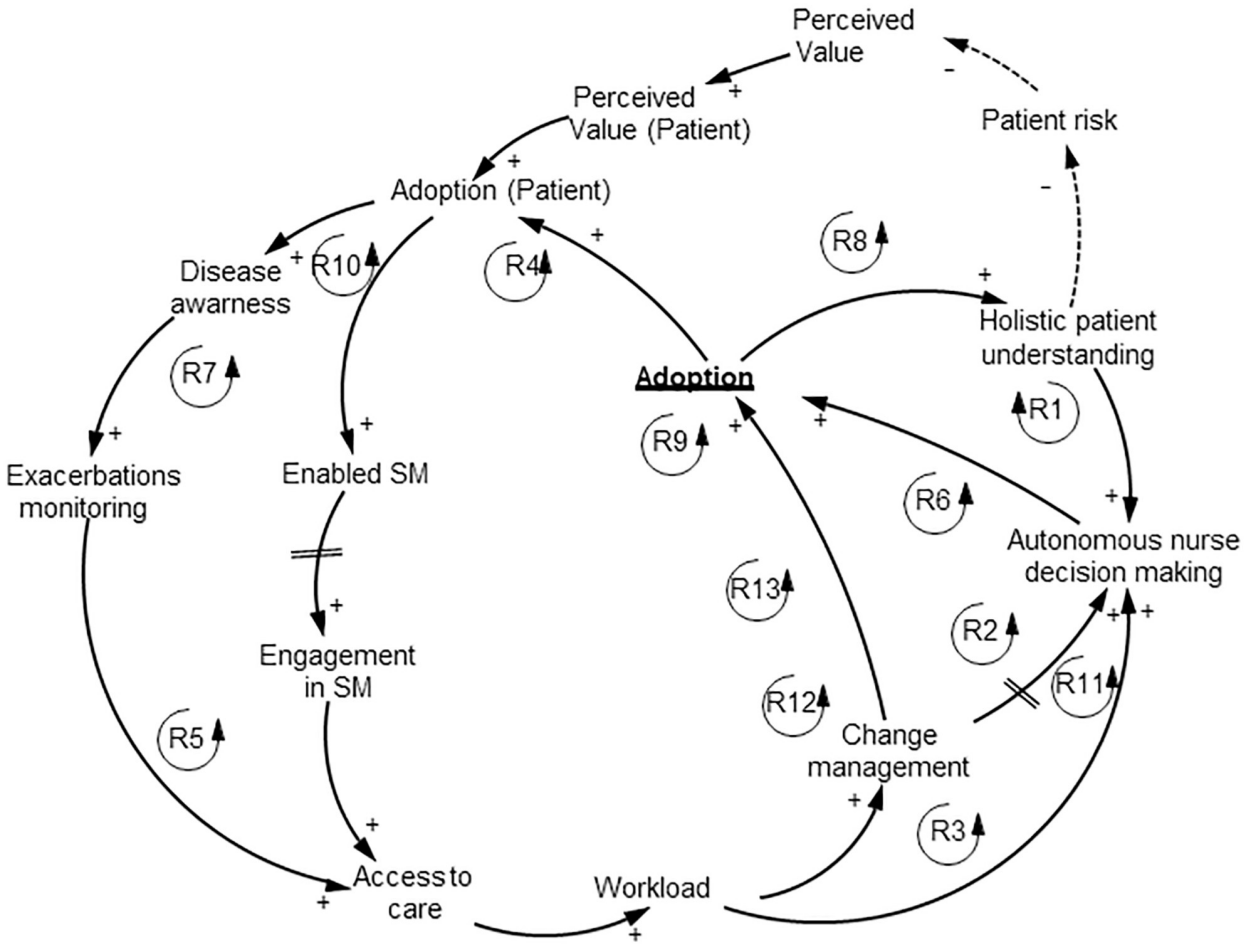
Nurse TH adoption feedback loops.

**Fig 2:**
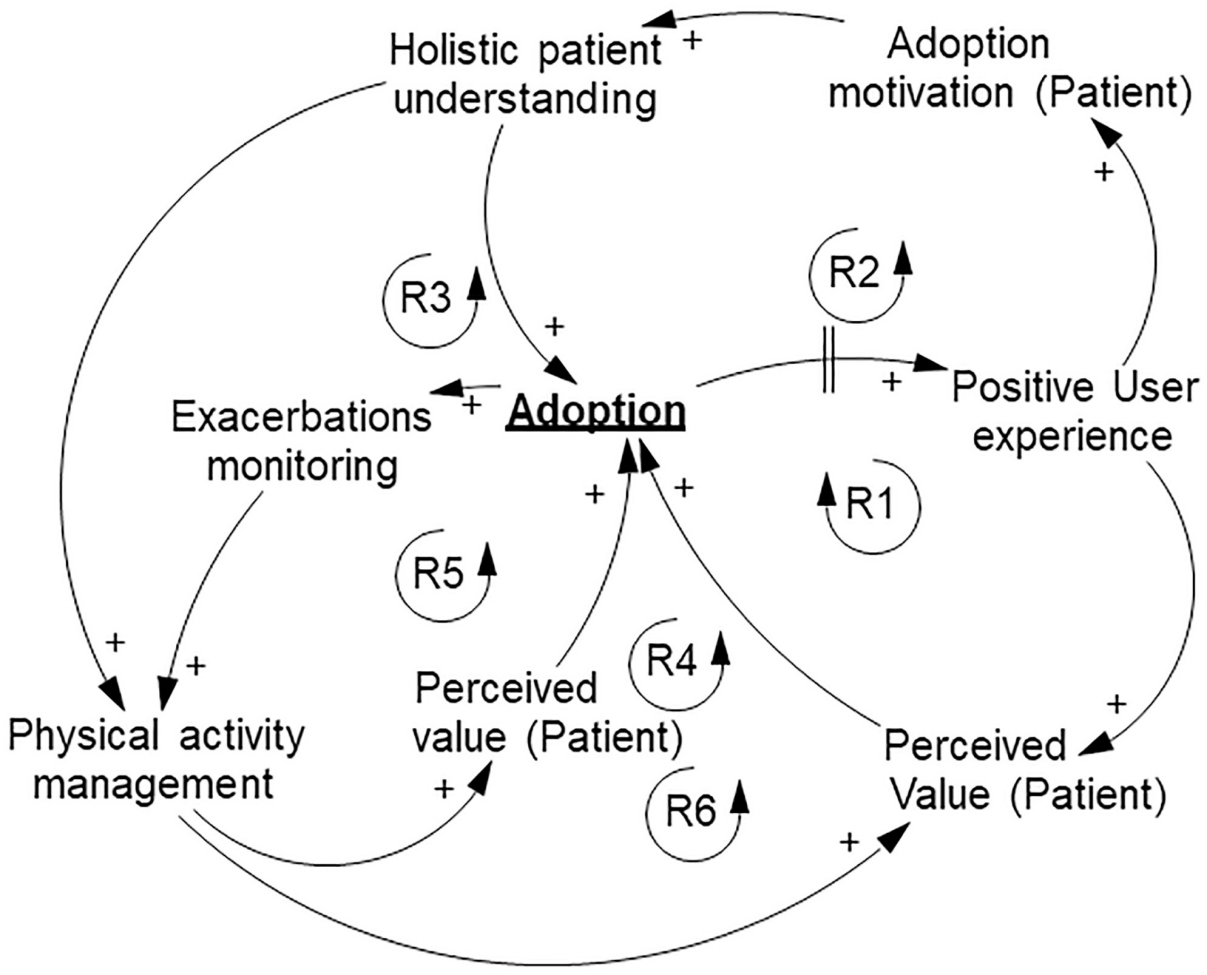
Physiotherapist TH adoption feedback loops.

**Fig 3:**
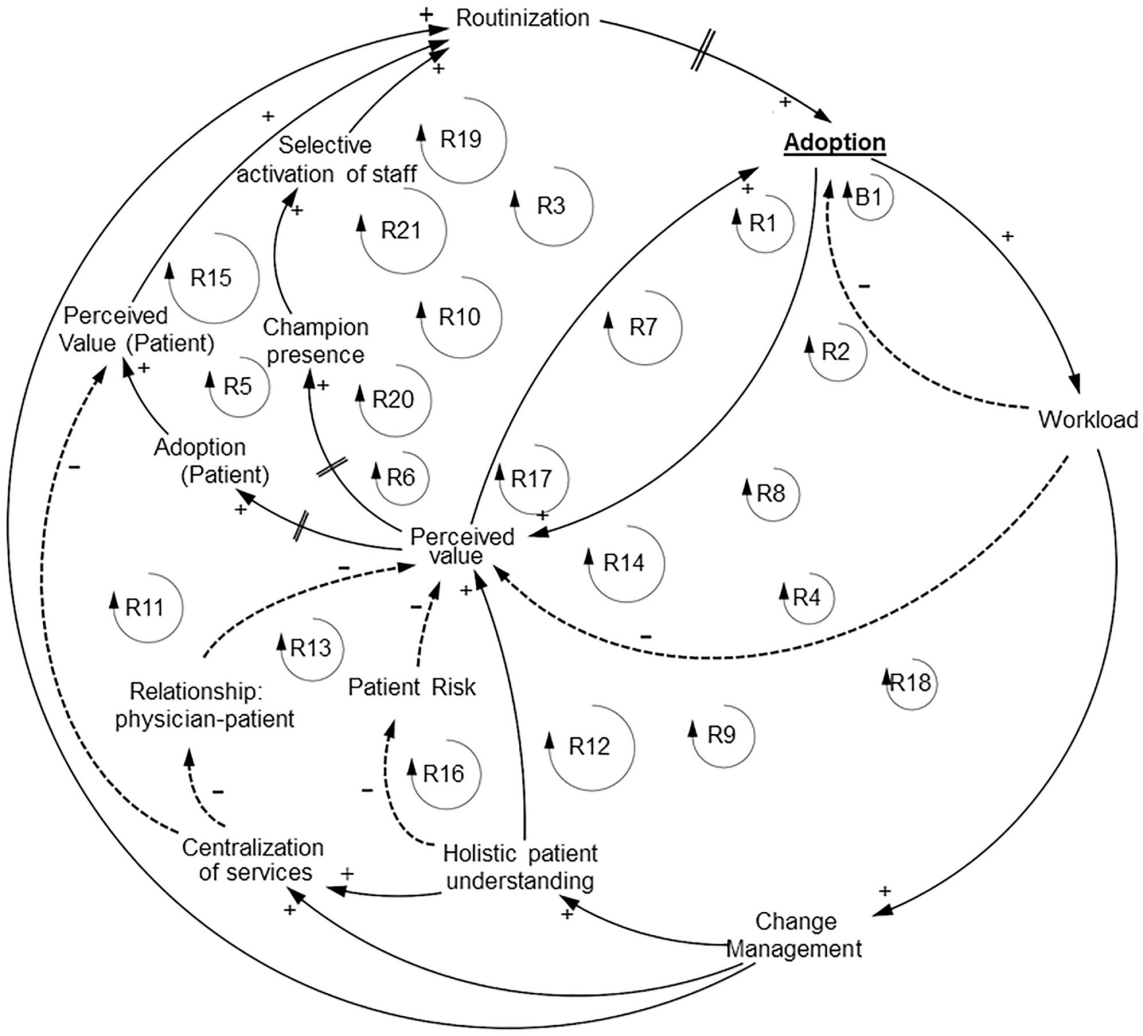
Physicians TH adoption feedback loops.

Tables representing cause and effect variables were created for each of three HCPs: physicians, nurses and physiotherapists. In the final figures (Figs 1–3), feedback loops were depicted which are focused on the main outcome of interest: “Adoption of TH” by the particular HCP (S3 Table), which is a part of the complex illustration. Variables used in the diagrams are described in Table 3.

**Table 3:** Description of variables used in causal loop diagrams.

Healthcare professional	Variable	Description
**Physician, Nurse**	Change management	Components that support TH service integration in care pathways
**Physician, Physiotherapist**	Holistic patient understanding	Considering the patient as a whole, e.g. understanding how a patient behaves when not in the care institution
**Physician**	Centralization of services	TH service support provided by a call center, which is not part of the institution providing care
**Physician**	Patient risk	Risks or adverse events related to the TH service usage
**Physician**	Relationship: physician-patient	Development or foundation building activities for patient-physician relationships
**Physician, nurse**	Adoption (Patient)	Accepting the use of TH service by patient
**Physician, Physiotherapist**	Perceived value (Patient)	Perceived values of TH services by patient
**Physician**	Champion presence	Leadership strongly advocating for TH adoption
**Physician**	Selective activation of staff	Activation of the right HCPs at the right time
**Physician**	Routinization	The regular use of TH service in care pathway
**Physician**	Workload	Poor time management due to task complexity, overburdened schedules, and increased workplace pressures
**Physician, Physiotherapist**	Perceived value	Perceived value of TH service by HCPs
**Physiotherapist**	Positive user experience	Positive user experiences when using TH services
**Physiotherapist**	Exacerbations monitoring	Constant patient monitoring to detect changes in parameters which indicate exacerbation(s)
**Physiotherapist**	Physical activity management	Personalization of physical activity based on the live health status of patient
**Physiotherapist**	Adoption motivation (Patient)	Intrinsic patient motivators to use TH services
**Nurse**	Autonomous nurse decision making	The degree of independent decision-making performed by a nurse when using TH
**Nurse**	Enabled SM	Tools and processes which enables the patient to use SM services
**Nurse**	Engagement in SM	A patient who in active in using SM services
**Nurse**	Access to care	A patient’s access to appropriate healthcare services
**Nurse**	Disease awareness	The degree to which a patient understands his/her condition and its severity

#### Nurse causal loop diagram

The validated nurse CLD contained 13 feedback loops to explain TH adoption for COPD management from the nurse perspective (Fig 1). Our variable of interest, “Adoption”, meaning nurse likelihood to adopt TH, was directly affected by the variable “Change management” and “Autonomous nurse decision making”.

Reported “Change management” comprised of: team support (or HCP being part of a multidisciplinary team); confidence building through TH training; establishing a plan for describing the setting and frequency of communication between HCPs; availability of resources such as time and extra team members for non-clinical tasks and new service coordination (i.e. training from nurse to patient). When “Change management” was present, nurses immediately adopted TH. If “Change management” was lacking, nurses were less likely to adopt TH in their everyday practice. In most cases, “Change management” was triggered by increased “Workload”.

“Workload” was caused by increased “Access to care”. “Access to care” had two features which increased “Workload”: 1) lack of scheduling, with patients calling when having a possible event and 2) triage procedure, which requires a lot of information and clinical evaluation skills. If nurse “Workload” was not increasing, it was unlikely that “Change management” was initiated. Increased “Access to care” occurred if a patient was able to recognize exacerbations, which was the result of increased “Disease awareness” or patient engagement in self-management (SM).

If a nurse had limited “Autonomous nurse decision making”, it was less likely that she would adopt TH. Considering “Autonomous nurse decision making”, nurse responsibilities varied between different healthcare settings from complete autonomy (Table 1) to generalist nurses which strictly followed protocols from physicians [56]. Based on our QES, stimulating “Autonomous nurse decision making” should not focus solely on increasing the scope of decision making. Other issues which may limit “Autonomous nurse decision making” are lack of policy guidelines for TH, limited access to physicians when the final clinical decision needs to be made, levels of clinical expertise, task distribution for non-clinical decision-making support by other staff, bad access to relevant clinical data. “Autonomous nurse decision making” may increase (with a certain time delay) after “Change management” introduction or “Workload” increase. From Fig 1, it is evident that “Autonomous nurse decision making” was the variable influenced the most by other variables (“Holistic patient understanding”, “Change management” and “Workload”). This is important information for future policy strategies, as this variable may be difficult to change.

Lack of “Holistic patient understanding” may lead to a decrease of “Autonomous nurse decision making”. For example, nurses observed that they need to better understand the patient’s character, which in most cases in COPD care is self-inhibiting. This means that patients with COPD do not like to bother anyone, even if they experience a disease crisis [57]. “Adoption” increased “Adoption (Patient)”, which created an indirect reinforcing loop for “Adoption”. The results from our QES indicated that nurses played a crucial role in patient adoption of TH in two ways: 1) screening for inclusion (e.g. perceived age influence, disease severity) and 2) communicating the “Value perceived” for TH adoption for COPD management.

#### Physiotherapist causal loop diagram

The physiotherapist CLD consists of 6 reinforcing feedback loops (Fig 2). The two variables “Holistic patient understanding” and perceived value by the physiotherapist “Perceived value” played a crucial role in TH adoption by physiotherapist, “Adoption”.

“Adoption” directly, with a time delay, affected “Positive user experience”. “Positive user experience”, contributed to “Adoption motivation (Patient)” and “Perceived value”. An increase in “Positive user experience” lead to an increase in “Perceived value”, with the opposite being true as well. If “Perceived value” decreased, “Adoption” was less likely. “Positive user experience” positively affected adoption likelihood by the patient, “Adoption (Patient)”. In the validation procedure, physiotherapists agreed that patient motivation decreases with time irrespective of how good a patient was engaged in the beginning of the intervention. Increased “Adoption (Patient)” resulted in greater “Holistic patient understanding”, because patients actively shared information and were involved in the decision-making process related to behavior change. If “Holistic patient understanding” increased, it positively affected two variables: “Adoption (Patient)” (insight in a patient’s everyday life, needed for further decision making, increased willingness to adopt) and “Physical activity management”.

“Physical activity management” was the main value and task for physiotherapists. Previously they had to rely on scheduled appointments with patients. However, TH allowed remote access to ascertain that an increase in physical activity was achieved. Moreover, the access to this data permitted personalization of physical activity management and allowed monitoring patients for signs of exhaustion. “Physical activity management” influenced “Perceived value” and “Perceived value (Patient)”. One of the not-evident variables contributing to “Adoption” was “Exacerbations monitoring”, meaning that it affects the “Physical activity management”. From our QES, it became clear that many decisions physiotherapists make are related to exacerbations of disease: procedure initiation, procedure continuation and success prediction based on exacerbation history.

#### Physicians causal loop diagram

The CLD for physicians (general practitioners (GPs) and pulmonologists) was the least saturated of the three different HCPs. However, the validation procedure was rich. This resulted in 22 feedback loops, out of which 21 were reinforcing (Fig 3). Three studies reported on pulmonologists, while the others five focused on GPs. The main variables contributing to TH adoption by physician (”Adoption”) were TH service sustainability (” Sustainability”), “Perceived value” and “Workload”.

”Adoption” increased when “Perceived value” increased. “Perceived value” is a very important variable for healthcare policy strategy as it was influenced by many other variables (N = 5) and difficult to change. “Perceived value” was affected by “Adoption”, which was in a closed, reinforced feedback loop. When “Adoption” increased, “Perceived value” increased as well, resulting in a backwards effect in the diagram. The opposite is true as well. “Perceived value” was as well affected by “Workload”, “Holistic patient understanding”, “Patient risk” and “Relationship: physician-patient”.

“Patient risk” decreased when “Holistic patient understanding” increased. According to our QES, “Patient risk” was mostly related with antibiotics use. Within a TH system, there is a lack of clear guidance for prescription of antibiotics. This may result in either over- or under-prescription, which is a risk to the patient. From the QES two risk types emerged: 1) related with intervention (i.e. not yet clear if SM is beneficial for COPD patients) and 2) related with the technologies supporting the intervention (i.e. not clear how to establish triage values in order to timely detect an exacerbation).

In our QES, “Change management” considers that TH integration in clinical practice should accommodate patient selection criteria, personalization of TH services according to patients’ needs and measurement frequency. “Centralization of services”, meaning service transfer to another clinical setting from the primary location, was detrimental to “Relationship: physician-patient” and it decreased perceived value for the patients. Increased “Workload” lead to increased “Change management” or decreased “Perceived Value”. “Perceived Value” affected “Champion presence” and adoption by the patient (“Adoption (Patient)”). If “Perceived value” decreased the likelihood of “Champion presence” in a healthcare setting was lower. Moreover, if “Perceived value” decreased “Adoption (Patient)” was less likely. Both processes occurred with a delay in time. “Champion presence” contributed to “Selective activation of staff”. As “Selective activation of the staff” increased, the likelihood of “Routinization” was greater as people would be partially responsible for the service scaling success. Increased “Routinization” over time can improve the process of “Adoption” for physicians who were hesitant in the initial stage of TH adoption. The perceived value by the patient increased “Routinization”. This is exemplified by this quote from one of the studies: “… we made this service so crucial for the patients and their families that it actually became irrevocable. I challenge the one who has the courage to stop it!” [49].

## Discussion

Adoption is a serious problem as interventions with positive clinical outcomes in small scale pilot programs are almost never recommended to be further implemented [58]. Documenting lessons learned in these pilots is crucial before TH projects can be scaled up successfully [59].

Starting with secondary literature analysis, relationships were documented that play a role in the adoption process, which were enhanced and validated by stakeholder interviews. The result was the basis for extracting the most important variables for future interventions. Our findings contribute to the field by proposing models where the entire process of adoption is visualized and by building awareness to problems associated with trial design. In the design of most clinical trials, the sole focus is on clinical outcomes. However, in the field of TH more attention should be paid to implementation. It is important to consider implementation strategies, monitoring the quality of adoption, identifying barriers and facilitators for participation.

Based on our analysis, following key variables were identified related to TH adoption for COPD management, which can contribute to future TH implementation strategies:

1. From the nurse perspective, “Autonomous nurse decision making” and “Change management” are crucial. It is important to understand how nurses make decisions and how autonomous these decisions can be. This variable is the most complex to address, as it has links to many other variables. Moreover, it is important to understand that nurses are key in the adoption process. Therefore, “Change management” processes should first address the needs of nurses.
2. From the physiotherapist perspective, the adoption process shows relatively low complexity. The focus is mainly on “Perceived value”. Therefore, evidence- based,” Positive user experience” and the ability of TH to support “Physical activity management” is important.
3. From the physicians perspective, the adoption process is the most complicated one.”Perceived value” is the key component. It is very difficult to affect through interventions, as it is closely related to other components. “Holistic patient understanding” and “Change management” are modifiable variables, influencing other variables in indirect ways. It is important to understand how “Change management” should be created from the physicians perspective and how to guarantee the process of “Holistic patient understanding” considering the limited time available to physicians.

While the analysis was done with separate CLDs for each stakeholder, they also depend and influence each other. For example, autonomous nurse decision making depends on the freedom provided by physicians. However, the discussed variables are used differently by the three different professionals. A good example is “Holistic patient understanding” relating to the decision-making process. Nurses use it to triage patients before consulting a physician. Physicians use it, albeit rarely, in combination with telemetric information for clinical decision making. The decision making process is not based on any single value, such as may be the case in diabetes [60] as COPD patients are multi-morbid, elderly, with a particular social profile [57,61]. Comparing the three CLDs, some variables are present in all of them, while some are unique for a particular HCP. For instance, “Centralization of services” and “Champion presence” were unique to physicians, while “Physical activity management” and “Positive user experience” were unique to physiotherapists.

It is important to note that there is currently no stakeholder responsible for self-management. The GOLD guidelines state that the physicians should take the lead in promoting patient self-management [1]. From our diagrams, the published literature and the stakeholder interviews it is evident that physicians “do not own” self-management service. In clinical pilots, nurses often manage self-management. This observation demands policy attention, as the clinical guidelines need to reconsider not only the efficacy of self-management, but also the self-conception of each stakeholder and their willingness to absorb and be responsible for new tasks.

In all CLDs, “Adoption by patient” or “Perceived value by patient” was a variable affecting other variables, indirectly leading to intervention adoption. This suggests that patients should not only participate in pilot studies, [43,45] but participate as well in change management procedures.

In the nurse and physicians CLDs, “Patient risk” contributed to the “Value perceived” by both professionals and was reduced by “Holistic patient understanding”. By understanding “Patient risk” related with TH service, the areas of the “Holistic patient understanding”, such as a patient character on which information needs to be registered, can be defined. For instance, in one of the trials, a nurse suspected that the patient was experiencing an adverse event, but he was not calling to register that. By knowing the patient’s character, which was shy and introvert, the nurse suspected that he is hiding details about his condition [54].

CLDs are a tool that helps to understand the problem in the healthcare settings [26]. It is a tool which is widely used in the field of public health and health policy decision making: participant retention in HIV prevention programs,[29] exploring trust building in vaccination [28] or understanding obesity prevention programs [39]. To our knowledge, our CLDs are the first example of how to model TH adoption in the field of COPD by different HCPs. Therefore, this work could be a pertinent starting point to enrich our understanding in the adoption of TH services for COPD patients’ care.

The article has several strengths and limitations. Considering its strengths, the qualitative evidence synthesis allows us to paint a picture of experience of HCPs, and is particularly robust in developing meta-aggregation of intervention adoption for policymakers makers [62]. The collaborative nature of the research permits to emphasize the most important variables by the different professionals; as it is the first article in the field, it contributes to future trial methodology allowing the use of the diagrams as initial common communication tool. The analysis has some limitations. The model is not quantified or tested in a particular setting. It is not meant to be exhaustive or definitive, rather an initiation document to start this paradigm in the field of TH. Due to the time constrains in the validation procedure, the patient perspective is currently lacking in our analysis. Moreover, evidence from interviews and ethnography published in the literature are at risk for bias as well.

## Conclusions

Our study offers an innovative approach to map and analyze the complexity of TH adoption process for COPD management. Adoption is different from various stakeholder perspectives. In order to improve quality of TH adoption, there is a need for multidimensional interventions which prioritizes needs of a particular stakeholder. Moreover, key variables were identified that require workable strategies to ensure success of telehealth services. Furthermore, teamwork capacity needs to be improved to accommodate self-management services in the care pathway of COPD patients.

Future research might include more sophisticated and computationally integrated methods, artificial intelligence and natural language processing, for a more automated analysis of the data. Moreover, the TH adoption process needs to be explored in patients, while GPs and pulmonologists should be explored separately.

## Supporting information

S1 Appendix Search algorithm. (DOCX)

S1 Table All initial variables, with a factor details and the supporting text. (DOCX)

S2 Table Main feedback loops considering different healthcare professionals. (DOCX)

S3 Table Validation description. (DOCX)

S1 Fig Complete CLD for three HCPs. (DOCX)
