# Supplementary material for: Developing a strategic understanding of telehealth service adoption for COPD care management: A causal loop analysis of healthcare professionals
Source: PLoS One. 2020 Mar 5;15(3):e0229619. doi: 10.1371/journal.pone.0229619 (PMC7058286; doi:10.1371/journal.pone.0229619)
Supplement: S1 Appendix — (DOCX) [file pone.0229619.s001.docx]

S1 Appendix. Search algorithm

#37

#35 AND #36

4,841,001

#36

'clinical':ti,ab AND 'trial':ti,ab OR 'clinical trial'/exp OR random* OR 'drug therapy':lnk

4,459

#35

#7 AND #34

523,610

#34

#23 OR #33

460,001

#33

#24 OR #25 OR #26 OR #27 OR #28 OR #29 OR #30 OR #31 OR #32

76

#32

'computer tablet':ab,ti

40

#31

'smart watch*':ab,ti OR 'smart-watch':ab,ti

389,936

#30

'computer*':ab,ti OR 'pc':ab,ti OR 'laptop*':ab,ti

12,801

#29

'handheld*':ab,ti OR 'hand-held*':ab,ti

2,174

#28

'mhealth*':ab,ti OR 'm-health*':ab,ti OR 'm health*':ab,ti OR 'mobile health':ab,ti

11,883

#27

'smartphone*':ab,ti OR 'smart-phone*':ab,ti OR 'mobile phone*':ab,ti

3,432

#26

'wireless communication'/exp

2,546

#25

'text messaging'/exp

3,695

#24

'mobile application'/exp

69,982

#23

#8 OR #9 OR #10 OR #11 OR #12 OR #13 OR #14 OR #15 OR #16 OR #17 OR #18 OR #19 OR #20 OR #21 OR #22

9,785

#22

'telemedicine':ab,ti OR 'tele-medicine':ab,ti OR 'tele medicine':ab,ti

104

#21

'connected health':ab,ti

2,950

#20

'tele-health':ab,ti OR 'tele health':ab,ti OR 'telehealth':ab,ti

3,819

#19

'ehealth*':ab,ti OR 'e-health*':ab,ti OR 'e health*':ab,ti

142

#18

'telenurs*':ab,ti OR 'telesupport':ab,ti OR 'telecommunic':ab,ti

12,702

#17

'telemetr*':ab,ti OR 'telemat':ab,ti OR 'telemonitor*':ab,ti OR 'tele-monitor*':ab,ti

2,326

#16

'videoconferenc*':ab,ti OR 'video group':ab,ti

2,477

#15

'remote monitor*':ab,ti OR 'remote consult*':ab,ti OR 'remote care':ab,ti OR 'councel':ab,ti

19,851

#14

'ambulatory monitoring'/exp OR 'self monitoring'/exp OR 'physiologic monitoring'/exp

2,388

#13

'videoconferencing'/exp

6,173

#12

'telephone telemetry'/exp OR 'remote sensing'/exp

7,741

#11

'teleconsultation'/exp

865

#10

'telemonitoring'/mj

5,586

#9

'e-counseling'/mj OR 'directive counseling'/mj OR 'patient counseling'/mj OR 'patient guidance'/mj

7,602

#8

'teleconsultation'/exp

138,528

#7

#2 OR #3 OR #4 OR #5 OR #6

41,308

#6

'chronic pulmonary disease':ab,ti OR 'chronic airway disease':ab,ti OR 'chronic lung disease':ab,ti OR 'emphysema*':ab,ti

64,647

#5

copd:ab,ti OR coad:ab,ti OR cobd:ab,ti OR aecb:ab,ti

14,518

#4

(chronic* NEAR/3 bronchiti*):ab,ti

167,304

#3

obstruct NEAR/3 (pulmonary OR lung OR airway OR airflow OR bronch OR respirat OR emphysema*)

100,381

#2

'chronic obstructive lung disease'/exp

4,841,963

#1

'clinical':ti,ab AND 'trial':ti,ab OR 'clinical trial'/exp OR random* OR 'drug therapy':lnk

(((((((((Pulmonary Disease, Chronic Obstructive[MeSH Terms]) OR ((COPD[Title/Abstract] OR COAD[Title/Abstract] OR COBD[Title/Abstract]))) OR (obstruct AND n3 AND (pulmonary OR airway OR airflow OR broch OR respirat OR emphysem))) OR (("Chronic pulmonary disease") OR "Chronic airway disease"[Title/Abstract]))) AND (((clinical[Title/Abstract] AND trial[Title/Abstract]) OR "clinical trials as topic"[mesh] OR "clinical trial"[pt] OR random*[Title/Abstract] OR "random allocation"[mesh] OR "therapeutic use"[sh]))) AND ((((((((((((((((((((((((("Telemedicine"[Mesh:noexp]) OR "Telemetry"[Mesh]) OR "Videoconferencing"[Mesh:noexp]) OR "Monitoring, Ambulatory"[Mesh]) OR "Monitoring, Physiologic"[Mesh:noexp]) OR (Remote monitor*[Title/Abstract] OR remote consult*[Title/Abstract] OR remote care[Title/Abstract] OR remote counsel*[Title/Abstract])) OR ((videoconferenc*[Title/Abstract] OR video group[Title/Abstract]))) OR (telemetr*[Title/Abstract] OR telemonitor*[Title/Abstract] OR tele-monitor*[Title/Abstract])) OR ((telehome*[Title/Abstract] OR tele-home[Title/Abstract] OR tele home[Title/Abstract]))) OR ((telenurs*[Title/Abstract] OR telesupport*[Title/Abstract] OR telecommunic*[Title/Abstract]))) OR ((eHealth*[Title/Abstract] OR e-health*[Title] OR e health[Title/Abstract]))) OR connected health[Title/Abstract]) OR (tele-health[Title/Abstract] OR tele health[Title/Abstract] OR telehealth[Title/Abstract])) OR ((telemedicine[Title/Abstract] OR tele-medicine[Title/Abstract] OR tele medicine[Title/Abstract]))) OR ((eCoach[Title/Abstract] OR e-coach[Title/Abstract] OR e coach[Title/Abstract]))) OR ((eLearning[Title/Abstract] OR e-learning[Title/Abstract] OR e learning[Title/Abstract]))) OR ((telemanagem*[Title/Abstract] OR tele-managem*[Title/Abstract]))) OR "Mobile Applications"[Mesh]) OR "Text Messaging"[Mesh]) OR ((smartphone*[Title/Abstract] OR smart-phone*[Title/Abstract] OR mobile phone*[Title/Abstract]))) OR ((smartphone*[Title/Abstract] OR smart-phone*[Title/Abstract] OR mobile phone*[Title/Abstract]))) OR ((mHealth*[Title/Abstract] OR m-health*[Title/Abstract] OR m health*[Title/Abstract] OR mobile health*[Title/Abstract]))) OR ((Handheld*[Title/Abstract] OR hand-held*[Title/Abstract]))) OR (((computer*[Title/Abstract] OR pc[Title/Abstract] OR laptop*[Title/Abstract])))) OR ((smart watch*[Title/Abstract] OR smart-watch*[Title/Abstract])))))) AND ((((perception*[Title/Abstract] OR experience*[Title/Abstract] OR acceptance*[Title/Abstract] OR satisfaction*[Title/Abstract] OR perspective*[Title/Abstract] OR view*[Title/Abstract] OR response*[Title/Abstract]))) OR ("Attitude of Health Personnel"[Majr]) OR "Patient Acceptance of Health Care"[Majr]))
