# Supplementary material for: Developing a strategic understanding of telehealth service adoption for COPD care management: A causal loop analysis of healthcare professionals
Source: PLoS One. 2020 Mar 5;15(3):e0229619. doi: 10.1371/journal.pone.0229619 (PMC7058286; doi:10.1371/journal.pone.0229619)
Supplement: S1 Table — (DOCX) [file pone.0229619.s002.docx]

S1 Table. All initial variables, with a factor details and the supporting text

| ***Doctor (GP or Pulmonologist)*** | | | | | |
| --- | --- | --- | --- | --- | --- |
| **Themes** | **B** | **F** | **Factors** | **Factor Details** | **Text** |
| **Adoption** | X |  | *Perceived Value* | Low perceived value by users (1) | In relation to videoconferencing, a doctor says, ‘I am able to hear in a telephone whether a patient’s breathing is in a bad condition. I am not sure how much extra I get informed by looking at a patient on a screen’.  Collaboration, tensions and boundaries between doctors and nurses  The collaboration between doctors and nurses is a source of frustrations. Rather than to follow highly standardised and labour-intensive procedures, the doctors think telecare ought to be practised according to the patient’s (not the nurses’) goals. The doctors worry that the routines seize the patients time without clinical results. They doubt whether videoconferencing after all relieve treatment and care, but support that close telecare follow-up in relation to the very sick COPD patients provides security. They stress that research is not yet able to document decrease in re-hospitalisation rates. Hence, they are concerned that politicians oversell the benefits of telecare. |
| **Organization** |  | X | *Champion* | Activation of the right persons at the right time (5) | (“I took those nurses and specialists that work better in my unit, those that I see having a deep sense of their practice”, case 2). |
|  | X |  | *Change Management* | Integration of TH into work practices (1, 4) | It is smart that the patient can submit measurements from home that they would otherwise have to go to the hospital to have done. What we do not know is for which patients this is most appropriate; how often to take measurements, and how it should be adapted to the individual patient. There are many unresolved issues. The technology is smart and it is here to stay. We just need to find out how to fit it into our work practice (14). Telecare ought to be flexibly arranged according to the individual patient’s needs and not by labour intensive standards, the doctors’ claim. They are worried that self-measurements take up time without results. |
|  | X |  | *Workload* | Poor time management (1, 13) | A doctor said, ‘I think it’s a shame for them when they run around and must have handed some information and need answers. Therefore, I help them. This is part of our treatment of our patients. The doctor should assist the nurses, but they are stressed’. Therefore, it is the day hospital doctor, who has to deal with telecare inquiries and since he/she has already recorded a full day program in OLC, he/she needs to be contacted in between other tasks. Not surprisingly, this means clinical decisions are postponed and that doctors act as bottlenecks.  ... where community teams are involved with our COPD patients, we get a lot more contact, a lot more calls, a lot of those are not appropriate in our eyes. A lot of buck passing. I think potentially that’s quite damaging to patient care. (‘Usual care’ GP, ID9) Professionals were divided on the effectiveness of cross-boundary working between telemonitoring and normal primary care services. While some felt that telemonitoring shifted pressure from hard- pressed GP and hospital services, for others interaction between services proved problematic because clinical roles and responsibilities were not delineated. While some GPs appreciated sharing communication about patients and working in partnership with telemonitoring professionals, others found the involvement of non- medical community-based telemonitoring professionals intrusive, unwelcome and unhelpful. |
|  | X |  | *Decision Making* | Inhibited or constrained ability to make decisions (15) or delegation (1, 13) | “I thought it would be more clear-cut. It didn’t occur to me it would be so difficult to work to a standard baseline. It’s a lot more complex than I thought it was going to be.” [GP 1] In contrast to the patients’ perception of the advantages of having a score to inform objective decision-making, clinicians recognised the limitations of a standardised threshold and described the importance of interpreting the scores in context. Workload had increased as telephone calls or, occasionally, visits were made to clarify the reality of the individual clinical situation when the score breached the threshold.  A doctor said, ‘I think it’s a shame for them when they run around and must have handed some information and need answers. Therefore, I help them. This is part of our treatment of our patients. The doctor should assist the nurses, but they are stressed’. Therefore, it is the day hospital doctor, who has to deal with telecare inquiries and since he/she has already recorded a full day program in OLC, he/she needs to be contacted in between other tasks. Not surprisingly, this means clinical decisions are postponed and that doctors act as bottlenecks. ... where community teams are involved with our COPD patients, we get a lot more contact, a lot more calls, a lot of those are not appropriate in our eyes. A lot of buck passing. I think potentially that’s quite damaging to patient care. (‘Usual care’ GP, ID9) Professionals were divided on the effectiveness of cross-boundary working between telemonitoring and normal primary care services. While some felt that telemonitoring shifted pressure from hard- pressed GP and hospital services, for others interaction between services proved problematic because clinical roles and responsibilities were not delineated. While some GPs appreciated sharing communication about patients and working in partnership with telemonitoring professionals, others found the involvement of non- medical community-based telemonitoring professionals intrusive, unwelcome and unhelpful. |
| **Service Design** | X | X | *Usability* | Poor usability or functionality (15), meets users’ needs (11) | “I want to be able to see the information for ... my 20 patients and be able to glance at it and not ... I mean to look at one person’s results must be – what eight different screens?” [GP2] The design of the clinician interface was perceived as constraining rather than enhancing the task of monitoring, as it took little account of clinical requirements or the limited time available for monitoring. “We found that the measured values that were accessible and visualised through graphics provided the patients with an overview of the development of their own symptoms, and they learn to act and foresee an exacerbation.” The healthcare professionals reported that they gave some of the patients more responsibility for managing their own disease, and the patients received a treatment plan consisting of prescriptions for penicillin and hormones and guidelines for what to do in case symptoms appeared. In this way, the patients became more active, changing their mind-set, and were able to perform self-management of their COPD. A doctor at the hospital remarked: |
|  | X |  | *Patient risks* | Risk to patients (15) | “But quite what effect all these antibiotics are going to have at the end of the line is a different story. It may be good if it keeps them out of hospital or if it makes them feel better generally, but I think ... these drugs are not without side effects.” [GP 1] In keeping with the patients’ assessment that the tele- monitoring was sensitive to changes in their condition, clinicians initially assumed that the marked increase in prescriptions for antibiotics and steroids reflected improved recognition of exacerbations, although the possibility that the threshold for triggering an alert may have been set too low was raised. Later, the risks and benefits of what was perceived as a substantial increase in prescribing became a concern.  Physiological measures were available to the clinicians, although their interpretation was not clear. Although oximetry was perceived as useful, lung function was considered to be unreliable and/or uninformative. |
| **Utility** | X | X | *Financial Structures* | No reimbursement for use of TH (5), institutionalization of financial incentives for use (5) | In case 1, the financing was institutionalized because the service became “too important to be stopped”. The head of the pulmonary unit stated that: “In a very short time, we made this service so crucial for the patients and their families that it actually became irrevocable. I challenge the one who has the courage to stop it!”. “having an institutional framework [i.e. the activation of the service of home hospitalization with a specific reimbursement] justifying the efforts made for the service with specific rewards, helped the service to survive”. In this regard, despite the professionals’ claim that financial incentives are not enough to motivate the professionals, an institutional frame is needed when the champion leaves the service. In case 2, the head of the pulmonary unit retired in 2013 and a physician stated that |
|  | X |  | *Relationship* | Limits the development or foundation building for patient-doctor relationships (13) | I think [it’s important that] the whole, holistic approach towards patients is not given away ... singling out a few values ... many kilometres away ... in a control room with various remote criteria ... but we are not quite sure what that means in the clinical context and how it is perceived by the patient. (‘Usual care’ GP, ID11) The proposition of the development of some form of regional ‘call centre’-type service reinforced one GP’s concerns about telemonitoring. The respondent, from a practice not participating in the trial, considered that the remote telemonitoring of patient data, undertaken outwith the parameters of established practice, compromised patient care. |
|  |  | X | *Self-Management* | Patients develop an understanding of themselves (4, 11) | It is smart that the patient can submit measurements from home that they would otherwise have to go to the hospital to have done. ==== Barrier from here ==== What we do not know is for which patients this is most appropriate; how often to take measurements, and how it should be adapted to the individual patient. There are many unresolved issues. The technology is smart and it is here to stay. We just need to find out how to fit it into our work practice (14). 4.4 Relationship between nurses and doctors  Telecare ought to be flexibly arranged according to the individual patient’s needs and not by labour intensive standards, the doctors’ claim. They are worried that self-measurements take up time without results. A doctor states:  Doctors agree that telecare in relation to very sick COPD patients provides safety, but they stress that research is not able to document decreased rehospitalization. Hence, doctors’ state that telecare is politically rather than clinically driven and they worry that politicians’ blindly oversell it (13, 14).  “We found that the measured values that were accessible and visualised through graphics provided the patients with an overview of the development of their own symptoms, and they learn to act and foresee an exacerbation.”  The healthcare professionals reported that they gave some of the patients more responsibility for managing their own disease, and the patients received a treatment plan consisting of prescriptions for penicillin and hormones and guidelines for what to do in case symptoms appeared. In this way, the patients became more active, changing their mind-set, and were able to perform self-management of their COPD. A doctor at the hospital remarked: |
|  |  | X | *Exacerbation* | Patients learn to recognize and react to exacerbation signs and symptoms (11) | “We found that the measured values that were accessible and visualised through graphics provided the patients with an overview of the development of their own symptoms, and they learn to act and foresee an exacerbation.” The healthcare professionals reported that they gave some of the patients more responsibility for managing their own disease, and the patients received a treatment plan consisting of prescriptions for penicillin and hormones and guidelines for what to do in case symptoms appeared. In this way, the patients became more active, changing their mind-set, and were able to perform self-management of their COPD. A doctor at the hospital remarked: |
|  |  | X | *Health Services* | Improves access to healthcare services (10) | It is smart that the patient can submit measurements from home that they would otherwise have to go to the hospital to have done. ==== Barrier from here ==== What we do not know is for which patients this is most appropriate; how often to take measurements, and how it should be adapted to the individual patient. There are many unresolved issues. The technology is smart and it is here to stay. We just need to find out how to fit it into our work practice (14). 4.4 Relationship between nurses and doctors  Telecare ought to be flexibly arranged according to the individual patient’s needs and not by labour intensive standards, the doctors’ claim. They are worried that self-measurements take up time without results. A doctor states:  Doctors agree that telecare in relation to very sick COPD patients provides safety, but they stress that research is not able to document decreased rehospitalization. Hence, doctors’ state that telecare is politically rather than clinically driven and they worry that politicians’ blindly oversell it (13, 14). |

| ***Nurses*** | | | | | |
| --- | --- | --- | --- | --- | --- |
| **Themes** | **B** | **F** | **Factors** | **Factor Details** | **Text** |
| **Adoption** | X | X | *Timing* | TH implementation must be sensitive to disease burden (2), hospital-initiated implementation of TH improves patient confidence (2) | “Round the ward would be good to encourage them to move, but if it was in the ward, the limitation would be those who can’t move without our help, and we don’t have enough nurses to go to bed 1 or 2, but it would help them regain confidence before going home.” [HC021]  Perspectives on timing “There’s nothing wrong with the timing it’s just the amount of information, I think, because when you’re not well yourself and to be bombarded with a lot of information to take on board you’re sitting there listening but how much do you take in.” [HC006] |
|  | X | X | *Implementation Support* | TH service installation challenges (10, 16), technical support improves implementation process (10, 17) | ‘More input and training initially. Easier points of contact when there is a problem ... time allowance ... more staff ... time for meetings/ support for training/time to do telehealth’  The nurses believed that the local NHS health services could do a lot to support the success of the service. More input and training, better communication plans, and regular telehealth team meetings were all factors, nurses believed, that would enhance and ensure the success of telehealth:  The patient’s husband wasn’t keen, and said almost straightaway that he was worried it would upset the regular phone, which he got especially for emergencies. Nurse 6 said they’d set up, and take it away if it was a problem. Nurse 7 also reassured him that it would make no difference to his ordinary phone. While they were setting up the equipment, the patient remarked ‘Aren’t you clever!’ and her husband added, ‘On top of this you’re engineers!’  Subsequent experience with the system both supported and confounded such views. Both nurses and researchers were surprised at some of the patients’ positive responses to the technology. The telehealth technology opened fundamental debates about professional values and status. The transporting and setting up of equipment were clear examples of potentially inappropriate professional activities, with one nurse describing feeling like a ‘carpet fitter’ after trying to hide cabling in a patient’s house. Another team member raised a counter- argument of even less desirable duties that nurses were sometimes expected to undertake. The following shows that the technology could have a real effect on how the nurses were seen by patients: |
|  | X |  | *Perceived Value* | Doubts value of TH for patient (16) or for supporting work practices (5, 16) | Nurse 8: Don’t think I would get as much job satisfaction just by seeing them on a television screen rather than seeing them at home. Attitudes The issue of satisfaction or dissatisfaction with the telecare system was a point of much discussion. Among the nurses, some felt satisfaction at mastering the use of new technology in this context, but a persistent theme remained that nurses felt that seeing patients face-to-face was more satisfying. Nurse 7: But I can see a role for tele-medicine, but not in the area that I am working in at the moment, but I can see where there would be a role for monitoring patients just baseline monitoring really, if you were monitoring someone just from their blood pressure point of view why do you have to go way out to their home just to chat to them and take their blood pressure, but I think it could be more beneficial with patients who are reasonably well, whereas our patients are acute and these patients should still be in hospital. So I can see it more for the well patients at the moment than for the ill patients. The nurses conceded that telecare did have a possible role in the management of long- term conditions, but were clear that they did not really believe the telecare service to be appropriate for their patient population and context. In doing so, they raised questions about the legitimacy of telecare as a form of service provision. In addition, some of the nurses also expressed the view that older patients as a group were less suitable for telecare, whereas the patients themselves seemed to believe that telecare was particularly useful for older people, living on their own. Nurse 11: There is always that threat isn’t there of new technology impinging on your role. I really enjoy the one-to-one relationships with patients that is where I feel my skills come to bear really, that is where I am most happy, in a one-to-one with the patient, teaching on a one-to-one basis, so I suppose there was that little bit to it and then I suppose there was the idea that it was more work on top of a busy job. Professional Issues For some of the nurses, the introduction of the telecare service was thought to have a negative impact on their professional identity and was even perceived as a potential threat. This was reflected by concerns about what widespread use of such systems might mean for nursing. For example, there was anxiety that increasing utilization of telecare systems might mean that fewer nurses would be required in the future, and thus by utilizing telecare technologies, the nurses feared that they might potentially be placing their future livelihoods in jeopardy. Furthermore, there were concerns that adoption of such systems would adversely affect, qualitatively, the nature of the work that nurses would be expected to undertake in the future and could have a detrimental effect on the holistic nature of nursing care. As stated by a nurse in case 3: “We cannot follow them all [all the patients affected by COPD at any stage]: we have selected a specific sample of conditions which require monitoring. Without this accurate selection, the service is not sustainable...and the patients are not going to use it [the service] if they don’t see that it's useful for them!” Telemedicine might not be equally effective for more specialized/acute care, where treat- ments should be continuously customized and tailored through multidisciplinary actions as the disease progresses |
|  | X |  | *Age* | Age of patient negatively influences decision to adopt TH (7, 17) | Nurse 3 talked about two [study] refusals last week. She said it was sometimes difficult because people of that age group [were] often unhappy at the thought of anything else [i.e. additional equipment]. Some haven’t used a nebulizer before.  The unpredictability of the equipment’s performance in the early stages inhibited conversational flow and made it difficult to present the technology confidently to patients. The negotiations between the nurse and researcher teams often raised the issue of the likely or Professional responses to a telehealth trial actual patient responses to the technology. The nurse in the extract below drew attention to patients’ other technological ‘responsibilities’, in which context even offering the telehealth option might upset nurse– patient relations:“quite old and elderly”, |
|  | X |  | *Low IT Literacy* | Perceived patient doubt in technology (7, 17) | The clinicians, however, were aware of “a small minority” who when “we show them the unit and how it works and reassure them with questions... (say) ‘oh no, I couldn’t cope with that’”. Age did not appear to be a barrier to the acceptance of a Telehealth monitoring service or of using the equipment. “a lot of them are quite frightened by technology”. The patient’s husband wasn’t keen, and said almost straightaway that he was worried it would upset the regular phone, which he got especially for emergencies. Nurse 6 said they’d set up, and take it away if it was a problem. Nurse 7 also reassured him that it would make no difference to his ordinary phone. While they were setting up the equipment, the patient remarked ‘Aren’t you clever!’ and her husband added, ‘On top of this you’re engineers!’ |
|  | X |  | *Patient Suitability* | Patients are missing out on using TH because they don’t fit narrowly defined user profiles (10) | ‘To allow the criteria to be widened, there are a lot of housebound patients who need this service, but to whom it is not available’ ‘To allow the criteria to be widened, there are a lot of housebound patients who need this service, but to whom it is not available’ ‘I have found it difficult to recruit patients due to the constraints set, there are a lot of patients who take a lot of GP/nurse time that would benefit, but as they have not had a hospital admission do not meet the criteria set’ They had no difficulties in recruiting patients, but the inclusion criteria were seen as too tight and prevented the service from being provided to a lot of patients who would benefit the most from it. ‘I have found it difficult to recruit patients due to the constraints set, there are a lot of patients who take a lot of GP/nurse time that would benefit, but as they have not had a hospital admission do not meet the criteria set’ They had no difficulties in recruiting patients, but the inclusion criteria were seen as too tight and prevented the service from being provided to a lot of patients who would benefit the most from it. |
|  | X |  | *Patient Motivation* | Low health status awareness impacts TH use (5) | n this regard, a nurse in case 2 reported a negative episode. The unit had a device available for use: “we did not have pending requests for the devices, so we decided to give it to a patient affected by a COPD at a moderate stage of the disease who, by the way, did not have a driving license and, as such, might have problems in reaching the hospital. We asked him to use the device once a week, although maybe he was not feeling that bad. As a result, the patient went home, put the device in a drawer and never used it! [...] the point was that he did not perceive the urgent need to monitor his clinical conditions”. About the patients, the cases showed that their acceptance is enhanced if the services target a specific need that they perceive as crucial for their personal wellbeing. |
| **Organization** | X | X | *Champion* | Forced by champion to train or take a TH role (10), confidence in champion’s positive attitude towards TH (5, 10) | nurse in case 3 said: “she [the head of the pulmonary unit] was convinced that the service was exactly what we needed. I wasn’t aware at that time, but I trusted my chief...and...well, now I can tell that she was right!”. In addition, the fact that the service champions in the three cases were the head of the pulmonary units conferred legitimacy to the introduction of the telemedicine service and made it easier to gather initial commitment from the professionals. ‘The service will not succeed and continue unless all GPs and... PCT are on board. The nurses believed that the local NHS health services could do a lot to support the success of the service. More input and training, better communication plans, and regular telehealth team meetings were all factors, nurses believed, that would enhance and ensure the success of telehealth: ‘My GP put me forward! ... happened very rapidly so I had to take over as I went along’.Organisational support and the role of health service authorities.Almost all the nurses had no say in the decision about joining the telehealth service, as their names were allocated by the GP. They saw it as being foisted on them with no consideration of their views of the service: ‘GP told us to attend training to set up telehealth ... I’ve no idea about it and had to start from scratch’. Organisational support and the role of health service authorities Almost all the nurses had no say in the decision about joining the telehealth service, as their names were allocated by the GP. They saw it as being foisted on them with no consideration of their views of the service: The nurses believed that the local NHS health services could do a lot to support the success of the service. More input and training, better communication plans, and regular telehealth team meetings were all factors, nurses believed, that would enhance and ensure the success of telehealth: |
|  | X | X | *Change Management* | Challenging to manage communication without a specific strategy (10), lack of procedure standardization (15), priorities for changes taking place are not established (3, 10), training support or sustains TH changes (10, 17) | ‘The training we had was simple ... straightforward ... comprehensive’ The training varied between different practices, as some nurses had 2 hours’ basic training and others had whole-day training. In general, they were satisfied with the training they received. For example: The patient’s husband wasn’t keen, and said almost straightaway that he was worried it would upset the regular phone, which he got especially for emergencies. Nurse 6 said they’d set up, and take it away if it was a problem. Nurse 7 also reassured him that it would make no difference to his ordinary phone. While they were setting up the equipment, the patient remarked ‘Aren’t you clever!’ and her husband added, ‘On top of this you’re engineers!’ ‘This could be supported in different ways ... having short regular meetings with the team would definitely help’ Holding more frequent 2-hour training sessions and arranging a twice-yearly meeting for telehealth nurses were suggestions given to improve the training: ‘This could be overcome by allocating more staff to deal with minor non-clinical alerts’ T e nurses suggested that having an additional team member, especially an administrator, to deal with non- significant triggers would overcome the overload issue ‘hMore input and training initially. Easier points of contact when there is a problem ... time allowance ... more staff ... time for meetings/ support for training/time to do telehealth’ The nurses believed that the local NHS health services could do a lot to support the success of the service. ‘We had a monitor at the surgery that had been waiting to be picked up ... for weeks ... I had sent emails to [the community nurse] ... but she replied she is no longer involved in the project’ The absence of a clear communication plan had negative effects on the service, as the nurses did not know how to communicate with the other service partners. One nurse described how she had unwanted equipment at her practice for a long time, not knowing whom to contact to have it taken away: “... there have to be good procedures in place for making sure that the information goes to whoever needs it ... without that you can have all the technology you like but it’s not going to be useful.” [Nurse Manager 1] In general the professionals’ perception of benefits outweighed the initial rearrangement of work practices to accommodate the monitoring. Healthcare professionals were cognisant of the responsibility to ensure timely and competent monitoring of the incoming information. ‘Due to so many changes in primary care recently, we are so very overloaded that telehealth new tasks are difficult to take on board’ Overall,all the interviewed nurses described their experience with telehealth to be positive. However, they were not satisfied with the resources available to run the service. The most common feature of the nurses’ experiences with telehealth was that it had heavily consumed their time. All the nurses agreed that updating the records and the triaging process was taking much longer than they had expected. Despite agreeing that telehealth was beneficial to the patients, the nurses reported that it affected their daily work and increased their already high workloads: “The project is evolving all the time. If you work on a ward, it is work [nursing] you have conducted for many years, and it is familiar and known, but here [at the TMC] it is a new way of working […] It is constantly changing. One live in such concentration because you experience things that are new. It is just how it is. It is not easy.” (Nurse 1). However, the TM intervention formed part of a newly project, and the recurring changes in the project  development and management of new work tasks was a challenge for all the nurses: ‘I think that telehealth has a place in ... health services but needs to be well defined and designed ... No it shouldn’t be a priority due to all the facts I mentioned earlier’ Owing to several factors, including cutbacks and lack of staff and funding, some nurses did not think that the implementation of telehealth should be a priority for local NHS health services. Others, however, believed that telehealth did have its place, but should be clearly defined: |
|  | X | X | *Decision Making* | Lack of precedence in policy for TH creates self-doubt for decision making (16, 17), limited access to doctors (1, 3, 4, 10), low levels of clinical expertise (3, 13), task distribution for non-clinical decision making can be supported by technical staff (10), improved access to relevant clinical data (1, 4), expands scope of decision making responsibility (1, 4) | ‘This could be supported in different ways ... having short regular meetings with the team would definitely help’ ‘stand professionally on tiptoe’. It is clear that the feeling and experience in relation to this responsibility adds new dimensions to the nurses’ portfolio of tasks. Thus, the real-location of tasks due to the telecare infrastructure demand that the telecare nurses’ knowledge encompasses a broad field of clinical expertise that makes it possible to act autonomously. In other words, the nurse must/ Thus, compared to the clinic nurse, the telecare nurse has a more autonomous position in terms of avoiding the patient’s re-hospitalization. The telecare nurse indeed needs more than in-depth knowledge of COPD. She needs to ‘our own patients’ Telecare reallocates the expertise because the nurse decides when to involve the doctor. This means for instance a new and important responsibility with regard to avoid re-hospitalisation of the patient. This makes them talk about the patients as. However, on-screen contact appears “non-real”, it is indeed “real” in the sense it can safe lives and it remediates professional tasks between doctors and nurses. In telecare, the nurse assumes responsibility for all tasks, and it is, as mentioned, the nurse who decides to involve a doctor. This transformation in terms of division of labour makes the nurses talk about.. Another nurse states: We have a huge responsibility. We see measurements from the patient every week and this makes us discover far more than if we only see the patient twice a year. We have discovered a heart condition several times. In that case, you need to respond to symptoms that are not only lung- related and we have a responsibility since it is us who assess when to involve a doctor (8). 4.3 New tasks for nurses  The telecare standards have implications for the engagement. In spite of the physical distance (and the impaired contact), nurses and patients meet in productive ways. Owing to the ongoing contact, nurses assume responsibility for responding to unforeseen health matters. In telecare, nurses more independently prepare and follow up on measurements than they do in OLC. Due to the ongoing contact, nurses assume responsibility for responding to unforeseen health matters. One nurse states: When we are in the clinic, we pull the patients to the doctor. In telecare, we set things in motion. If they need a blood test, x-ray or saliva sample, I order it (10). The telecare standards have implications for the engagement. Nurse 3: Oh dear well, I...you have brought up the problem of the risk problems, I would have said it is quick and if the patient’s well then you know there is not a problem its fine. But now when I think about the risks that I didn’t think about, missing things, I mean I always thought about missing signs and symptoms but you know, the court side of it, yes I don’t know how I would be fixed in court if I had to go and tell the judge well she looked fine from the neck up. Safety  A repeated concern of the nurses was that of risk and whether, in a court of law, a telecare service would be deemed safe or not.  This mapped onto the question of relational integration, raising problems of confidence in using the system. Medico-legal issues were a recurrent theme, and the reasons under- pinning these concerns are highlighted by the following statement: Nurse 1 feels they would be losing credibility in the patients’ eyes if not visiting, especially in the first four days. ‘I would feel very uneasy about not visiting them’. ‘Our role is to visit them’. Perhaps would use it in the patient’s second week. But not putting registration on the line in a court of law if patient deteriorates — therefore wouldn’t do a phone call in first four days. Better than a standard phone call, but not as good as a visit. The nurse underlines the risks involved by noting that the police can break into people’s homes on their responsibility. ‘We can’t afford to be held up and say you didn’t visit the patient’. Professional security  The technology was sometimes seen as undermining nurses’ professional security and credibility, and there was some concern about its potential to replace nurses. The issue of the risks to patients was also raised, and some nurses felt that patients, who tended to be in an older age group, would find it difficult to cope with the technology. One saw relying on virtual contact in the acute early stages of illness as particularly risky: The question repeatedly posed by nurses is 'How do we get faster and better contact with the doctors’. With regard to this, a nurse took a picture of the doctors’ roster. The photo shows the telecare column in the doctors’ roster and that this column is consistently uncompleted. This means telecare is not assigned to a specific doctor, despite such a task exists in clinical practice and as a column in the roster. This tells us that in spite of the strong need for lung doctors in telecare, their contribution are to some degree formally abandoned. There are tensions among doctors and nurses regarding conditions for contributing to telecare. While nurses have ample time, doctors have full programmes at the clinic. Thus, doctors understand telecare as a nurse project, where they occasionally help. Not surprisingly, the big question for nurses is. According to a nurse, ‘You have to come crawling . . . I’ve gone all day waiting for a response from a doctor . . . when you want to get hold of a doctor you need to beg: could you just help me? ... some of them respond ... who has telecare today?’ Therefore, it is the day hospital doctor, who has to deal with telecare inquiries and since he/she has already recorded a full day program in OLC, he/she needs to be contacted in between other tasks. Not surprisingly, this means clinical decisions are postponed and that doctors act as bottlenecks. Others miss colleagues and some simply state, ’It can be too trivial’ or ‘it may be too silent’. Nursing in telecare, in relation to OLC, clearly demands more independent decision-making. It makes a mentionable difference in the delegation of tasks that in telecare, the nurse decides when to involve a doctor. In OLC, the doctor is always there and takes the clinical decisions. A nurse states “you often say, let’s talk about that next time! And you know very well that there will be no next time” The clinic work is busy. Typically, in the clinic, a doctor and many patients are waiting. Moreover, owing to the 15-min consultation with unknown patients, there is not much time for conversation. In summary, the COPD patient in the clinic sees the doctor and nurse twice a year. In telecare, the patient sees the nurse 12 times a year (on screen) and the doctor once a year. This is in opposition to what is often the case in OLC, where the nurse works under a constant time pressure. There are types of conversations with patients that the nurse never starts, because they know they do not have the time to finish them. A nurse says, ‘In telecare, the nurse summarises and decides what to do, unfortunately when this involves medication, which is often the case; the absent doctor must be involved, who then has to be found out there’. During the 20 observed videoconferences, I only witnessed one doctor talking virtually on screen with a patient for a few minutes. This left the impression of a close-knit professional boundary between doctors and nurses. The doctor repeated many questions the nurse had just asked. This demonstrates adherence of the well known division of work as practiced in OLC: in OLC the patients always meet both nurse and doctor and they meet the nurse first. The doctor summarises and decides what to do. “I must be honest. I am the one that has worked here for the shortest time, and I think that it has been difficult to get into this way of working.” (Nurse 3). Environmental aspects, such as the function and organisation of TM work, influenced the reasoning and decision-making processes. The nurses’ abilities to interact with the environment were based on their TM experiences. The nurse employed for the shortest time found the work more challenging: .. it’s about putting everything into perspective with a COPD patient. It’s knowing that somebody may normally have sats [oxygen saturation] of 85, ... I think somebody who is an administrator may look at that and panic ... It’s knowing the baseline and a bit more about the disease ... (Nurse, secondary care, ID22) Practitioners considered clinical expertise important in interpreting and contextualising telemonitoring data relative to the individual’s ‘normal’ readings over time. Just as nurses have a phone which telecare patients can call, doctors have a phone which nurses can call. It is not picked up! The doctors rely on us reaching them, if we really need them. We depend on them, but they do not depend on us (10). There are tensions among doctors and nurses regarding conditions for contributing to telecare. “When I am alone at work I have to decide (concerning a patient) what I should or should not do… one feels very alone.” (Nurse 2). Moreover, the nurses managed the TMC alone, which restricted collaboration between the nurses, physicians, and other healthcare professionals when making decisions |
|  | X | X | *Workload* | Unscheduled tasks are time consuming (10), increase stress levels (14), triaging and readings are time consuming (10), improved distribution of tasks (1) | A nurse estimates ‘to have time available gives you job satisfaction and it really makes a difference’. Another pivotal aspect in telecare that supports strong relationships is the fact that videoconferencing with patients is booked flexibly and one hour apart. In relation to normal controls in OLC, this means there is plenty of time to interpret measurements and evaluate the implications of the measurements in relation to the patient’s everyday life. Typically, in OLC, a medical doctor is waiting and so are perhaps twenty patients. ‘Patients attended A&E as they can’t get GP appointment when asked to do so by the telehealth service ... more GP support is required’ Finally, the limited number of GPs dedicated to telehealth led to difficulty in getting appointments when the telehealth system askged the patient to contact their GP. As a result, the patients went to accident and emerency (A&E), causing unnecessary incidents ‘Our problem is time ... I have found it time- consuming ... especially checking the readings and follow-up triaging’ Overall, all the interviewed nurses described their experience with telehealth to be positive. However, they were not satisfied with the resources available to run the service. The most common feature of the nurses’ experiences with telehealth was that it had heavily consumed their time. All the nurses agreed that updating the records and the triaging process was taking much longer than they had expected. Despite agreeing that telehealth was beneficial to the patients, the nurses reported that it affected their daily work and increased their already high workloads: ‘Finding the time to fill forms in, getting patients in surgery for 30–40 min is difficult, as limited number of appointments and only myself doing telehealth’ In addition, a shortage of staff working on the service was another aspect that the nurses highlighted as a barrier. They considered telehealth services to be an ‘overload’ on their busy timetables, yet they did manage to fit it in with their day-to- day work: ‘GP services already very busy and short-staffed ... most of fall-back work goes onto nurses who are the ones who daily oversee telehealth ... but we manage to cope with it ... I do the reading during my lunch break!’ In addition, a shortage of staff working on the service was another aspect that the nurses highlighted as a barrier. It has certainly increased the pressure of the job because you are very much aware, with the alerts coming through daily. For example I didn’t get to check my email until the after- noon, so if there is an alert coming in the morning and you haven’t seen that until the afternoon . . .there is a little bit of pressure there thinking, you need to check them in the morning. It’s not always feasible to do that. A number of technical implementation challenges were identified. Aside from the difficulties with the first supplier, time to install equipment and home broadband connections was underestimated, utilizing more staff resources and resulting in project delays. Most respondents had concerns about impact on their current and future workload. The current workload was described as manageable; however, there were concerns expressed if the number of COPD patients using telehealth was to increase. The home COPD Pods affected community and district nursing workload in particular. This may impact upon future ‘skillset workability’. “It is frustrating when you go all in. Telemedicine should be fast and simple, and almost all we do is sit fiddling with technical problems”. (Nurse 3). Consequently, video consultation follow-up was often replaced by telephone calls, meaning that the relevant visual information used for the reasoning and decision-making processes were difficult to implement. Furthermore, the recurring technical problems affected the nurses’ view towards their work and led to increasing frustration. The focus group interview revealed these frustrations with one nurse |
| **Service Design** | X | X | *Usability* | Ineffective interoperable systems (3, 10) or redundancies (3), concerns for error prevention due to confidentiality (16), intuitive design (1, 15) | Several nurses state that it motivates them when telecare patients take responsibility for their own body. As one nurse puts it: ‘What is promising in telecare is when the patient sends “Red” measurements or “Yellow”, you just respond. You know the patient and you simply call and ask how they are. Later, we have a video meeting where I can observe how the patient is. You have a relationship to the patient. The patients take responsibility. There is an educational aspect to it’. Nurse 4: Yes because if there were people there, we don’t know who is in the room with them see, we didn’t know whether there was the next door neighbour there, the milkman had called in for a cup of tea, and we don’t know, unless they are sitting behind the patient looking into the camera we had no idea who was in the room. We always used to say to them because we had to say for security reasons that when we made the telephone, we would say are you ready, are you happy for us to see you and whatever, then you can press the button and we can make the visual link. The nurses also expressed concerns about privacy and confidentiality issues using the telecare system. For example: ‘The system can’t communicate with our system, I have to enter the same data in two places ... it is extra work ... it doesn’t produce any useful report ... it just takes data in and not out’ The triage system was not integrated with the practice software, which led to duplication in the data entry. In addition, the system had limited options for reports and data analysis tools, which made it difficult to produce evidence on the efficacy for both nurses and health services authorities. “I need to combine the [system] information with something. I cannot look at the CDSS recommendation alone. I need to see the whole picture, the [patient’s] history, everything.” (Nurse 1). The data collected, including nursing documentation, which was based on medical records, medical histories, and the system recommendations, were diverse and multifaceted. Mapping of cues and combining clinical data contributed to a clinical overview, assisting interpretation of pattern changes specific to individual patient’s symptoms and disease development. “The nurses are constantly handling two different software systems on two different computers. They spend much time searching for clinical information, leading to double entries. It seems ineffective and unnecessary, but such is the system.” (Fieldwork observation). |
|  | X | X | *User Experience* | Not entirely fulfilled by patient-technology interaction (4), lacking a purpose (3), increased job satisfaction (1, 3, 14, 17), perceived attitudes of patients’ find TH appealing (10) | Nurse 3 noted that telecare was ‘More than a phone call isn’t it?’ The nurses seemed to think it would be better to see them, and they see the nurse, as part of assessment rather than ‘just’ speak over the phone. Less commonly, the technology was seen to offer potential advantages: Such views formed a debate about the technology’s potential value, because at this stage there was little first-hand experience of using the equipment in practice. ‘Patients were excited about it ... easy to enrol if they met the inclusion criteria’ Patients’ engagement. The patients engaged well with the service and were easy to recruit once they matched the inclusion criteria. Despite having some patients who saw telehealth as a chore, and required more focused follow-up, the nurses described the patients’ attitudes regarding using telehealth as being positive. ‘The patients that were chosen were very happy and not a problem’ Patients’ engagement. The patients engaged well with the service and were easy to recruit once they matched the inclusion criteriae. ‘Extremely positive ... they have loved it ... are happy to be kept an eye on’. Patients’ engagement The patients engaged well with the service and were easy to recruit once they matched the inclusion criteria. Despite having some patients who saw telehealth as a chore, and required more focused follow-up, the nurses described the patients’ attitudes regarding using telehealth as being positive. According to another nurse, telecare constitutes an important incentive to apply technology. She says,’the fact that the technology is useful to the patients stimulates me to learn more about the technology and bring myself in a situation where I can do more with technology and thus become more effective as a nurse’. A nurse states, ‘I think it’s amazing that you do not get disturbed. Normally, seven nurses sit together in one office. It disturbs. You are not interrupted here. It is nice’. Most nurses are satisfied with this more responsible position, however in front of an unexperienced nurse, I observed, the demands on knowledge and expertise lead to insecurity. Most nurses appreciate the calmness and expertise in telecare. “You get a different relationship with patients [using TM and video conferencing] … somewhat closer, and it is much more personal.” (Nurse 2). the nurses found some of the aspects of TM work challenging, they also found it interesting, and the environment of working alone provided time and space to manage the patients regarding care planning, information gathering and consultations. In addition, time could be devoted to thoroughly conducting the reasoning process without distractions. Similarly, the TM setting enabled the development of strong nurse-patient relationships beyond the patient’s health, such as their personalities and life histories: A nurse estimates ‘to have time available gives you job satisfaction and it really makes a difference’. Another pivotal aspect in telecare that supports strong relationships is the fact that videoconferencing with patients is booked flexibly and one hour apart. In relation to normal controls in OLC, this means there is plenty of time to interpret measurements and evaluate the implications of the measurements in relation to the patient’s everyday life. Typically, in OLC, a medical doctor is waiting and so are perhaps twenty patients. Patients get much more appropriate and timely management of their condition doing telehealth because they’re not so invisible’. Despite initial concerns from some staff, home monitoring had good ‘interactional workability’, and the technology was described as ‘straightforward’ and easy to use. |
|  |  | X | *Holistic Patient Understanding* | Improves understanding of the patient as a whole (1, 3, 4) | “You get a different relationship with patients [using TM and video conferencing] … somewhat closer, and it is much more personal.” (Nurse 2). However, while the nurses found some of the aspects of TM work challenging, they also found it interesting, and the environment of working alone provided time and space to manage the patients regarding care planning, information gathering and consultations. In addition, time could be devoted to thoroughly conducting the reasoning process without distractions. Similarly, the TM setting enabled the development of strong nurse-patient relationships beyond the patient’s health, such as their personalities and life histories: Another nurse states: We have a huge responsibility. We see measurements from the patient every week and this makes us discover far more than if we only see the patient twice a year. We have discovered a heart condition several times. In that case, you need to respond to symptoms that are not only lung- related and we have a responsibility since it is us who assess when to involve a doctor (8). 4.3 New tasks for nursesThe telecare standards have implications for the engagement. In spite of the physical distance (and the impaired contact), nurses and patients meet in productive ways. Owing to the ongoing contact, nurses assume responsibility for responding to unforeseen health matters. In telecare, nurses more independently prepare and follow up on measurements than they do in OLC. Due to the ongoing contact, nurses assume responsibility for responding to unforeseen health matters. |
|  | X |  | *Patient Risks* | Nature of the patient is self-inhibiting (15), status of patient is permanently oriented around being a patient resulting in a sickness loop (12) | “There’s one lady that says ‘No’ [on the questionnaire] every day. But she’s probably the worst patient on it. Her chest is terrible. I know she’ll be struggling but she answers ‘No’. She says ‘I don’t like to bother anyone’.” [Practice Nurse 1] In contrast to the patients’ perception of the advantages of having a score to inform objective decision-making, clinicians recognised the limitations of a standardised threshold and described the importance of interpreting the scores in context. Workload had increased as telephone calls or, occasionally, visits were made to clarify the reality of the individual clinical situation when the score breached the threshold. I feel it reinforces a sick model for the patient... the patient would see themselves as very unwell on a daily basis because they’re constantly focussing on their disease state. (Nurse, secondary care, ID24.) Professionals also worried that patients ‘fixated’ on oxygen saturation levels as a health indicator and that this reinforced a ‘sick model’. |
|  | X |  | *Logistical Issues* | Incorrect or unnecessary equipment provided (10, 16) | ‘Equipment is provided as a set, patients taking extra unneeded equipment ... then miss these readings on daily basis’ The peripheral devices were available as a whole package, which meant that unnecessary devices were delivered to patients, leading to a large number of missed readings. Also, unclear instructions made the devices (such as thermometers) difficult for some patients to use: ‘A lot of patients do not use thermometers correctly’ Nurse 1: One of us would have to go out fit it up which did take some time, because you would be behind couches and everywhere, because if the patient had nowhere really to put it...It took a while to fit up...On the team personally it took one of us, for sometimes an hour or more, to fit it. And . . . it wasn’t a nurse’s job to fit it. We were being taken away from the patients, just fitting it up, it just wasn’t a nurse’s job at all. Equipment Issues Nurses were initially enthusiastic about the telecare service, but once they had experience of it in use, their enthusiasm faded. The first category, equipment issues, covered a number of areas, including: installation; picture and sound quality; reliability of physiological monitoring; and issues of trust or confidence in the equipment. Although the telecare units used the patient’s ordinary phone lines and were expected to be relatively easy to set up, from the nurses’ perspectives this still posed a number of difficulties: |
|  | X |  | *Technical challenges* | Equipment challenges (3, 16, 17), problems with voice quality or delay (1, 4, 16, 17) or visual quality is poor (1, 4, 16) | Nurse 4: And also of course the communication, there was a delay on the voice thing, it was like a two-radio which was really, really disconcerting at times . . . So quite often you were talking over each other...and it got really, really confusing. Nurses perceived that the system in use rendered the clinical communication process of any consultation more difficult and less effective. The system thus had limited interactional workability. Nurse 10: Sometimes you didn’t see the patient clearly at all . . . depending where the patient had the system set up in the home . . . You could see her sitting on the stairs but you wouldn’t know what colour she was, what shade of blue or grey or anything, you couldn’t make out that from her and I found it really difficult to observe her breathing because the picture quality wasn’t good. The nurses did not believe that the time-consuming and technical task of installing the equipment was nursing work, and argued that it would have been preferable for a technician to do this. But also, with regard to picture and sound quality, the general view was that there was great scope for improvement. Their confidence in the system was reduced because of what they saw as poor-quality communication. The following comments illustrate this: A nurse clarifies: I do not feel I can use my clinical gaze in the same way when I see a patient behind a screen. You are confronted with slightly lower sound quality, and the colours are not always optimal. I can only really see their face [. . .] I am not able to listen to their breathing like if they were sitting across from me. It is impossible to notice if they have cold fingers when they put the oxygen metre on. You can only ask whether this is the case. I cannot observe whether they have blue lips and nails (8). 4.2 Arrangement of the nurse–patient contact Technical problems lead to invisible work, even at the end of NetKol. Frequently, the devices in the patients’ homes do not work. Thus, the nurses contact a technician. Initially, they had to call the service provider’s general customer support and wait! Later, Fasin, the failure report system was developed, but this was demanding in use. Not until the end of the pilot project, the nurses contacted knowledgeable customer supporters directly. OPUS, the journal system, was on several occasions inaccessible for more than 1 h. Not surprisingly, it annoyed the nurses to to see patients without updated knowledge. Most importantly, Open Tele had persistent technical problems. The picture and sound stuttered or the system closed down when the patients’ transferred the bluetooth oxygen saturation figures. Then the nurse had to call again to note them manually. There were also problems with clinical assessment. ‘I do not feel I can use my clinical gaze in the same way when I see a patient behind a screen. You experience lower sound quality, and the colours are not always optimal. I can only really see their face ... I am not able to listen to their breathing as I would if they were sitting across from me. It is impossible to notice if they have cold fingers when they put the oxygen meter on. You can only ask whether this is the case. I cannot observe whether they have blue lips and nails’. The nurses tell stories about cameras in a patient’s home pointing at the ceiling or about patients not being appropriately dressed during video conferences. Rather than being respectless, I sensed the nurses thus managed situations characterised by impaired contact. A nurse concisely states what is at risk. Talking about a current patient, nurse 4 reported a poor picture — ‘You wouldn’t know if she was dead or alive, except you can hear her giggling’. She wondered whether it was a problem with the underground telephone cable. Everything else worked today, but some days nothing has worked. She said the speech delay was very off-putting, and that it was hard to get a follow-on conversation going. The data came through from the patient. His pulse is over 120 and the nurse is uncertain how to interpret this — Is it usually that high? Is the patient anxious? . . . At first his blood pressure cuff isn’t inflating, so (checking with researcher 1) nurse 2 asks him to check the connection in the back of the machine . . . . She tells the researcher she can see the patient’s wife putting his cuff back on. Eventually it inflates and the reading comes through. The temperature reading also takes two attempts. Nurse 2 feels it’s a bit high, but nurse 4 (also in the office) thinks it’s within normal limits, and that she wouldn’t worry about it. After the readings are all through, nurse 2 asks the patient ‘So how are you in yourself?’ He says he’s been for a test that shows he’s got ‘no carbon in him’ (she relays this to the researcher, as the speaker-phone is not being used), which means his lungs are clear. She tells him he’s done very well. She asks about his nebulizer — he’s on four, and she asks if he feels okay to reduce to three. She suspects from a previous contact that he’s stressed by using the equipment, and asks him whether he wants to carry on in the study. This work involves seeking a general patient assessment (‘So how are you in yourself?’), giving encouragement and negotiating changes in drug treatment. An advocacy role is also enacted in the question of whether the patient was finding participation too stressful. The extract illustrates the kind of ‘technical’ difficulties encountered, and shows how the nurse, the patient and his wife worked to achieve successful use of the technology. Researcher: How did you overcome . . . the fact that the temperature thing wasn’t working? Nurse 7: You then have to ask them key questions, how are you feeling, do you feel cold, do you feel feverish, you know, do you look flushed, do you feel flushed things, other physical findings, what colour is the sputum, things that may indicate or they may say to you I really don’t feel very well today, and you think is it just that they are having a bad day or are they running a fever are they brewing another infection, so the temperature is quite an important one really, particularly if you are dealing with the elderly during the winter. The nurses seemed to feel that telecare visits gave them extra ‘work’, in that, apart from installation, additional training was needed for patients, and if there were technical difficulties, e.g. temperature probe not working or something similar, then they had to spend more time taking a detailed history to compensate for the absence of clinical data, as this quotation demonstrates: This therefore described how use of the telecare system would slow down the clinical interactions, rendering them less efficient. Problems here were related to both interactional and skill-set workability. “Technical failures occurred often. These were perceived as a burden on both the patient and nurse and the technical factor became the urgent problem. Technical problems overshadowed the patients’ health problems, and the reasoning and decision making processes were disturbed. The TM nurses were tiring and sometimes postponed fixing the problem. It became a dilemma of time and resources.” (Fieldwork observation). In addition, technical problems often interrupted the FU before addressing clinical problems. Consequently, the focus on medical urgency was displaced by the focus on technical difficulties. Fieldwork observations revealed that almost half of the daily conversations with the patients involved attempts to find a solution to technical problems. Once technical difficulties were resolved, patients were often tired and needed rest. Technical difficulties were mostly related to tablet malfunctions including the application or 3G/4G coverage, which wasted time and interrupted the nurses’ reasoning  and decision-making processes: |
| **Utility** | X | X | *Relationships* | Inferior for building relationships or trust between nurse and patients (1, 4, 16, 17), improves access to clinical staff (1, 14), increases quality of communication (1, 17), develops patient openness (1, 3, 4, 10) | “in real life” This nurse claims that on-screen contact is inferior to the physical encounter. Telecare impairs the ability to provide an assessment as long as there is no opportunity to also see the patient Nurse 1 feels they would be losing credibility in the patients’ eyes if not visiting, especially in the first four days. ‘I would feel very uneasy about not visiting them’. ‘Our role is to visit them’. Perhaps would use it in the patient’s second week. But not putting registration on the line in a court of law if patient deteriorates — therefore wouldn’t do a phone call in first four days. Better than a standard phone call, but not as good as a visit. The nurse underlines the risks involved by noting that the police can break into people’s homes on their responsibility. ‘We can’t afford to be held up and say you didn’t visit the patient’. Professional security The technology was sometimes seen as undermining nurses’ professional security and credibility, and there was some concern about its potential to replace nurses. The issue of the risks to patients was also raised, and some nurses felt that patients, who tended to be in an older age group, would find it difficult to cope with the technology. One saw relying on virtual contact in the acute early stages of illness as particularly risky: Professional security The technology was sometimes seen as undermining nurses’ professional security and credibility, and there was some concern about its potential to replace nurses. The issue of the risks to patients was also raised, and some nurses felt that patients, who tended to be in an older age group, would find it difficult to cope with the technology. One saw relying on virtual contact in the acute early stages of illness as particularly risky: Nurse 8: Don’t think I would get as much job satisfaction just by seeing them on a television screen rather than seeing them at home. Attitudes The issue of satisfaction or dissatisfaction with the telecare system was a point of much discussion. Among the nurses, some felt satisfaction at mastering the use of new technology in this context, but a persistent theme remained that nurses felt that seeing patients face-to-face was more satisfying. Nurses described the most valued aspects of their work as ‘seeing patients face to face in their own homes’. One said ‘I love my work. I love going out to them.’ Telehealth consultations were presented as a barrier to ‘getting to know’ patients in the wider sense. However, reservations about the technology were generally less visible during observed patient contact, when the nurses provided encouragement and reassurance. Here, the need to maintain patient confidence in the service, to be ‘positive for the patients’, was given priority. Nurse 6: I think you physically need the patient in front of you, because there are a lot other issues, . . . I didn’t like speaking on it (videophone), . . . you can sit, it’s when you start talking to them and getting more of a rapport with them that they open up and tell you things and you have all their inhalers in the house, you can sit down and go through technique and do all the stuff like that. Nurse–Patient Relationship Researcher: Do you think that, [with the telecare], you said everything you wanted to say to the patient? Nurse 2: No. Not at all. No. I felt as if I couldn’t, it was no different to a phone call to be fair, no different to what I would say on a phone call, you can’t express yourself the same on the phone or even on a tele-med, you need to be with that person.The nurses expressed the view that using telecare was not ‘as good’ as home visiting and that it affected their relationship with patients in a negative way.This was not a view shared by patients. The nurses perceived that the telecare system had adverse effects on the professional–patient relationship. They also believed that using the telecare system reduced the chances of fully achieving important objectives of their clinical interactions, such as obtaining a full and accurate history and providing effective educational input. This again demonstrated deficiencies in the interactional workability of the system. From the nurses’ perspective, signal and image quality was a problem. But the style and content of their communication was also different when using the telecare system as compared to face-to-face visiting. They felt that there were real deficiencies in communication using the telecare system. The following exchange exemplifies the type of limitations in communication described by the nurses. |
|  |  | X | *Healthcare Services* | Improves access to healthcare services (10, 16) | Patients get much more appropriate and timely management of their condition doing telehealth because they’re not so invisible’. Despite initial concerns from some staff, home monitoring had good ‘interactional workability’, and the technology was described as ‘straightforward’ and easy to use. Regular interaction between nurses and patients took place: Several nurses state that it motivates them when telecare patients take responsibility for their own body. As one nurse puts it: ‘What is promising in telecare is when the patient sends “Red” measurements or “Yellow”, you just respond. You know the patient and you simply call and ask how they are. Later, we have a video meeting where I can observe how the patient is. You have a relationship to the patient. The patients take responsibility. There is an educational aspect to it’. One nurse for instance explains ‘the patients open up more, so it’s easier to help them because you get a closer cooperation, than if you only see them every six months for 15 minutes’ The nurse sees the patient every month via screen whereas the doctor sees the patient every year. The continous follow-up on weekly measurements provides the nurses with a unique understanding of the patients’ health condition. ‘Telecare is about pedagogy. I will observe the patient and guide [him or her] and the patient has the opportunity to contact me. They cannot do that in OLC. The patients learn to observe themselves and their symptoms and how they are’. The question of professional effectiveness as a nurse is interesting in terms of telecare’s implications for nurses’ tasks, discretion and relationship to doctors. Nurse 3 noted that telecare was ‘More than a phone call isn’t it?’ The nurses seemed to think it would be better to see them, and they see the nurse, as part of assessment rather than ‘just’ speak over the phone. Less commonly, the technology was seen to offer potential advantages: Such views formed a debate about the technology’s potential value, because at this stage there was little first-hand experience of using the equipment in practice. One nurse explains “they open up more, so it’s easier to help them because you get a closer cooperation than if you only see them every six months for 15 min” Because the nurses attend telecare on certain weekdays, they often meet the same patients. Continued follow-up on measurements makes strong relationships and gives the nurses a unique understanding of the patients’ condition. “You get a different relationship with patients [using TM and video conferencing] … somewhat closer, and it is much more personal.” (Nurse 2). However, while the nurses found some of the aspects of TM work challenging, they also found it interesting, and the environment of working alone provided time and space to manage the patients regarding care planning, information gathering and consultations. In addition, time could be devoted to thoroughly conducting the reasoning process without distractions. Similarly, the TM setting enabled the development of strong nurse-patient relationships beyond the patient’s health, such as their personalities and life histories: ‘[Telehealth] builds good relationships between patients and their care teams’ The nurses believed that telehealth could be beneficial to the patients as it empowered them and stopped them calling 999 inappropriately. On the other hand, for these benefits to be realised, the nurses felt, the appropriate patients should be included and frequently reviewed: |
|  |  | X | *Self-Management* | patients’ decisions in self-management (7),patients are enabled to be activated for self-management (1, 9, 12, 14), increasingly aware of their disease (1, 9, 14) | The clinicians noted patients “became more aware of their (oxygen levels), they became more aware of how breathless they actually get in normal activity and what is a daily variance for them. So whereas in the past they’ve just had this chest condition that’s all encompassing they are beginning to focus and find out about what is happening”... “Rather than just thinking ‘I‘m always breathless’, well you know you could say in what context are you always breathless... and it helps them start to understand their condition a lot better”. Several nurses state that it motivates them when telecare patients take responsibility for their own body. As one nurse puts it: ‘What is promising in telecare is when the patient sends “Red” measurements or “Yel- low”, you just respond. You know the patient and you simply call and ask how they are. Later, we have a video meeting where I can observe how the patient is. You have a relation- ship to the patient. The patients take responsibility. There is an educational aspect to it’. ‘Telecare is about pedagogy. I will observe the patient and guide [him or her] and the patient has the opportunity to contact me. They cannot do that in OLC. The patients learn to observe themselves and their symptoms and how they are’. ‘What it’s done is it’s helped patients to think, “Okay, right, well the weather’s bad, yes, I understand now why my oxygen levels are low, because today it’s a really muggy day.” So it’s almost reiterating, helping them to see more visually what’s going on for them.’ (Community matron, Cornwall). Telehealth as an empowering or burdensome influence on patients Telehealth can empower the patient. The vast majority of nursing participants viewed telehealth as a revolutionary and potentially beneficial change in the delivery of health care, as long as it was presented to patients as an optional rather than essential part of their healthcare plan, supplementing rather than replacing traditional health care. Most described it in terms of personal benefits to patients, who, they believed, gained new knowledge about their condition: In their experience, telehealth patients were becoming adept in recognising a correlation between their health behaviours and their biometric signs, for example the effect of smoking and exercise on the patient with COPD, or the effect of diet on the patient with diabetes: ‘They understand their long-term condition more now. If a COPD patient felt that they were getting an exacerbation, if they were coughing a little bit more, if their chest was a bit tight and they could see their SpO2s going down, then straight away they would be off to the GP themselves. And probably they would be phoning us and saying “Oh I’ve been to my GP because I’ve got a chest infection.” Whereas if they are left to their own devices, they didn’t have that equipment, they would, they wouldn’t know and probably two or three days down the line their chest infection would be getting worse.’ (Community matron, Newham) They considered that patients were becoming expert in understanding their condition, leading to behavioural changes and often to improvements in, or stabilising of, their condition and quality of life: I think they are more aware of their health. . .they are thinking about ‘how do I feel today?’ They’re more aware of their illness and what to do and what not to do as the symptoms arise. Most interviewees felt home telehealth monitoring for COPD was appropriate for community nurses to manage and could be integrated into established working patterns, demonstrating a high level of ‘relational integration’. The ‘skill set workability’ was appropriate for community nursing teams, providing an opportunity to increase their knowledge of COPD. Most felt it was appropriate for rural patients to be monitored at home and that telemonitoring promoted self-management: I think the whole concept of self-management is new. . . we’ve all been brought up with the idea that you’ve got an NHS that’ll look after you: just do what the professionals tell you because they know best, and now we’re unpicking that and people are having to learn. . . (Nurse, ID20) Professionals acknowledged the challenges in adapting established attitudes, behaviors and practices to address the challenges of telemonitoring-supported self-management. |
|  |  | X | *Comorbidity* | Helps to detect comorbidities (1, 4) | Another nurse states: We have a huge responsibility. We see measurements from the patient every week and this makes us discover far more than if we only see the patient twice a year. We have discovered a heart condition several times. In that case, you need to respond to symptoms that are not only lung- related and we have a responsibility since it is us who assess when to involve a doctor (8). 4.3 New tasks for nurses. The telecare standards have implications for the engagement. In spite of the physical distance (and the impaired contact), nurses and patients meet in productive ways. Owing to the ongoing contact, nurses assume responsibility for responding to unforeseen health matters. In telecare, nurses more independently prepare and follow up on measurements than they do in OLC. Due to the ongoing contact, nurses assume responsibility for responding to unforeseen health matters. A nurse states, ‘We have repeatedly discovered that the patient had a heart condition’. The nurse as both accountable and autonomous Because of the continuous contact and the strictly standardised system of accountability based on repeated follow-up on measurements, the nurses meet an array of complicated clinical quesions. The weekly measurements that the patients submit often provide opportunity to identify symptoms that the nurse, or any other professional, would not otherwise discover. |
|  |  | X | *Physical Activity* | Provides behaviour change programs for exercise (2) | Views on technology “I think it would be a good prompt to remind them to move and exercise, and it might help them focus, I think it’s a good idea.” [HC026] |
|  |  | X | *Patient Engagement* | Perceived patient confidence increased (10), focus (2), | ‘It gives them confidence and stops them ringing 999 so quickly ...Would be good for patients if they are appropriate for telehealth’ The nurses believed that telehealth could be beneficial to the patients as it empowered them and stopped them calling 999 inappropriately. On the other hand, for these benefits to be realised, the nurses felt, the appropriate patients should be included and frequently reviewed: Views on study design “I think that’s a really good idea. It will give them something to focus on. It may give them a bit of drive, a bit of focus, and it encourages people to become active, cuz obviously a lot of these patients who get very breathless and they can’t walk too far, so that will prompt them just to do small bits of exercise.” |

| **Physiotherapist** | | | | | |
| --- | --- | --- | --- | --- | --- |
| **Themes** | **B** | **F** | **Factors** | **Factor Details** | **﻿Text** |
| **Adoption** | X | X | *Perceived Value* | Indecisiveness or lack of interest in TH (6), desired impact and support for TH (2, 6) | PHTs from 15 practices mentioned they would be interested in using the intervention, provided that it proved effective and that the helpdesk would remain available. Two practices stated that they would not be interested in using the intervention. Two practices were unclear on this matter. Ability to see patterns in PA (to monitor exacerbations.Tool to start a conversation about PA with the patient…. “They are looking forward to getting home and getting back to some kind of normality, but hopefully they are thinking to themselves, I don’t wanna do that again anytime soon, so what’s gonna help me not do that again, and this study could help with that.” [HC011] |
|  | X |  | *Age/Disease severity* | Perceived difficulties due to age of patients (2, 6) | Nine PHTs mentioned that there were differences among the patients with regards to digital skill level. Personal instruction on the use of the intervention was deemed important, especially for older users. Views on technology “Some patients, maybe the older ones possibly who are just not very technologically savvy, they may struggle with something like this, and maybe the ones who are more end stage COPD, they might not see the point or be too overwhelmed possibly.” [HC022] |
|  | X |  | *IT Literacy* | Lack of technical capabilities and awareness (2, 6) | Views on technology “Some patients, maybe the older ones possibly who are just not very technologically savvy, they may struggle with something like this, and maybe the ones who are more end stage COPD, they might not see the point or be too overwhelmed possibly.” [HC022] Nine PHTs mentioned that there were differences among the patients with regards to digital skill level. Personal instruction on the use of the intervention was deemed important, especially for older users. |
|  | X |  | *Motivation* | Diminishing interest in TH (6), patients unwilling to accept disease status (12) | I think some people will never accept the fact that they have a chronic lung disease that will never get better. . . therefore there is no motivation to self-manage. . . (Community respiratory physiotherapist, telemonitoring team, ID18) The data obtained from professionals presented markedly different perspectives. Often they observed that patients actively deferred responsibility for the medical management of their condition to healthcare professionals during periods of ill health. Some attributed this limited engagement in self-management to a lack of acceptance of COPD as a chronic disease state and questioned whether the presence of telemonitoring technology in the home would be sufficient on its own to facilitate (re)consideration of self-management attitudes and behaviors among patients. |
| **Organization** | X |  | *Workload* | Burden of tasks is high (6) | However, reported use was low due to time constraints. The additional log­in was considered tedious, and the PHTs mentioned that a website that could be incorporated into their usual patient software would be better. |
| **Service Design** | X | X | *Usability* | Concerns of privacy (6), difficulty using services or interventions (6), services fulfilled user needs (6, 13) | Two PHTs mentioned that privacy is an important consideration when using eHealth. Six PHTs mentioned that the use of the mobile phone, as well as continuously wearing the mobile phone, was considered troublesome for one of their patients (but not for the rest of their patient group). Six PHTs mentioned that some of their patients had trouble sending text messages as a result of the small keyboard or overlooked the possibility. However, reported use was low due to time constraints. The additional log­in was considered tedious, and the PHTs mentioned that a website that could be incorporated into their usual patient software would be better. PHTs believe that the use of the intervention should be individually tailored to each patient. PHTs believe that face­to­face contact every 2­3 months is necessary, in addition to monitoring from a distance. I think it’s probably best if [telemonitoring is done by] people that are dealing with the patients every single day and have that bit of a relationship with them ... people are more trusting and it is something a bit more personable for the patient. (Telemonitoring physiotherapist, ID29) Knowing the patient Relationship continuities were also important to the professionals. For many practitioners, effective telemonitoring could only be accomplished by ‘getting to know’ the patient. PHTs suggested that patients should have the option to indicate if they were having a bad day and, subsequently, that their daily PA goal would be adjusted accordingly.…. All interviewed PHTs considered the website to be explicit and user­friendly. They used it to view PA data, adjust PA goals, and send text messages. |
|  |  | x | *Holistic Patient Understanding* | Appreciating the patient as a whole person (6, 8) | Nine PHTs mentioned that there were differences among the patients with regards to digital skill level. Personal instruction on the use of the intervention was deemed important, especially for older users. "Now the patients look like real human beings”. Telemedicine training gave the staff the opportunity to gain insight into a patient’s everyday life because they saw the patient dressed in his or her own clothing and in his or her own home and surroundings: a physiotherapist with experience in telemedicine training commented |
|  | X | X | *User Experience* | Failed to sustain patient motivations for use (6), Using the service was a positive experience (6, 13), well-received by patients (6) | PHTs mentioned that patients’ and their own motivation to use the intervention diminished over time… PHTs mentioned that the app was explicit and user­friendly for their patients…. People have really liked our service ... Yes, we’ve crossed a few boundaries and a few people have thrown their toys out the cot. “I do that, why are you coming and doing that?” I think we’ve managed it quite well by going “We can all work together”. (Telemonitoring physiotherapist, ID18)… |
|  | X |  | *Patient risk* | Patient reliance upon intervention (12) | I had one patient on the machine and he hasn’t been in hospital for a year. . . He accredits that to our service and the machine, and is petrified that when the trial ends that machine will be taken away from him, because it has become his life line. . . he’s become dependent and believes he should be getting phone calls on a regular basis. (Community respiratory physiotherapist, telemonitoring team, ID18.) However, after reflecting on interactions with patients in the intervention group during the trial, professionals described high levels of adherence to telemonitoring. Many considered that patients’ access to data combined with increased accessibility of telemonitoring services increased both the depth and frequency of communication between them and patients. Whilst this was often considered beneficial in terms of supporting early intervention and preventing exacerbations and subsequent hospitalization, many expressed concern about creating dependence on the technology and/or practitioner support, particularly among patients with severe COPD. |
| **Utility** | X | X | *Relationship* | Centralization limits relationship between PHT and patients (13), continuous TH monitoring by the same person improves relationships with patients (13) | I don’t know what to make of the fact that people consider sending it to call centres for monitoring ... I find that quite extraordinary really because you need to know the patients as a professional to be able to do the monitoring effectively ... (Telemonitoring physiotherapist, ID19) Professionals and patients both opposed the vision of centralised regional models of telemonitoring provision. They considered integration of telemetric provision with local practitioner services preferable to centralised ‘call centre’-type provision, emphasising the value of relational continuity over cost benefits associated with centralisation. I had one patient on the machine and he hasn’t been in hospital for a year. . . He accredits that to our service and the machine, and is petrified that when the trial ends that machine will be taken away from him, because it has become his life line. . . he’s become dependent and believes he should be getting phone calls on a regular basis. (Community respiratory physiotherapist, telemonitoring team, ID18.) I think it’s probably best if [telemonitoring is done by] people that are dealing with the patients every single day and have that bit of a relationship with them ... people are more trusting and it is something a bit more personable for the patient. (Telemonitoring physiotherapist, ID29) I think it’s probably best if [telemonitoring is done by] people that are dealing with the patients every single day and have that bit of a relationship with them ... people are more trusting and it is something a bit more personable for the patient. (Telemonitoring physiotherapist, ID29) I think it’s probably best if [telemonitoring is done by] people that are dealing with the patients every single day and have that bit of a relationship with them ... people are more trusting and it is something a bit more personable for the patient. (Telemonitoring physiotherapist, ID29) Knowing the patient Relationship continuities were also important to the professionals. For many practitioners, effective telemonitoring could only be accomplished by ‘getting to know’ the patient. |
|  | X |  | *Financial Structures* | No incentive for use of TH (6) | There were questions regarding the financing of the intervention. PHTs were concerned that they would not be paid by health care insurers because monitoring is not seen as a consultation; therefore, expenses cannot be claimed. |
|  |  | X | *Self-Management* | Perceived that patients are enabled to activated in their self-management (12) | Self-management would mean that they were able to see their warning signs independently. . . They would be able to act upon those warning signs appropriately and seek help, either by taking their anticipated care meds [medicines] that they have at home, or seeking help via a GP, or seeking help through the teleservice team. (Community respiratory physiotherapist, telemonitoring team, ID19.) Healthcare professionals considered ‘patient empowerment’ resulting from self-management as beneficial, so long as patients exercised personal responsibility over health and lifestyle choices within medically acceptable parameters. For many, the function of self-management was primarily to support patient adherence to medical advice and treatment. Professionals outlined the importance of formalized self-management training and support to engender medically compliant attitudes and behaviors in patients. They placed emphasis on their role in supporting ‘patient preparedness’ to self-manage through the provision of self- management plans, through coaching and advice, and through the dispensing of information materials. In this context, self- management was sometimes described as something ‘done’ to patients. |
|  |  | X | *Exacerbation* | Perceived likelihood of usefulness for patients to prevent exacerbations (6) | The PHTs believe that the eHealth intervention may be useful in preventing relapses and subsequent repeated PR. |
|  |  | X | *Physical Activity* | Personalization of physical activity based on live health status of patient (6) | PHTs mentioned that the intervention provided them insight into the objective PA data of their patients outside the clinical setting, whereas previously they had to rely on the account of the patient. This was regarded by them as a major advantage. It also enabled them to see patterns in PA. Nine PHTs mentioned that the ups and downs in the PA of patients with COPD are important to monitor in light of exacerbations. The data can be used to start a conversation with the patient about their PA level and to give them insights and tips. One PHT mentioned that his patients learned how far they needed to walk to reach their PA goal during the intervention period and continued to do so after the study ended. |
|  |  | X | *Patient-engagement* | Increased patient interest in health status and related activities to reduce hospitalization (2, 12) | “They are looking forward to getting home and getting back to some kind of normality, but hopefully they are thinking to themselves, I don’t wanna do that again anytime soon, so what’s gonna help me not do that again, and this study could help with that.” [HC011] I had one patient on the machine and he hasn’t been in hospital for a year. . . He accredits that to our service and the machine, and is petrified that when the trial ends that machine will be taken away from him, because it has become his life line. . . he’s become dependent and believes he should be getting phone calls on a regular basis. (Community respiratory physiotherapist, telemonitoring team, ID18.) However, after reflecting on interactions with patients in the intervention group during the trial, professionals described high levels of adherence to telemonitoring. Many considered that patients’ access to data combined with increased accessibility of telemonitoring services increased both the depth and frequency of communication between them and patients. Whilst this was often considered beneficial in terms of supporting early intervention and preventing exacerbations and subsequent hospitalization, many expressed concern about creating dependence on the technology and/or practitioner support, particularly among patients with severe COPD. |

B: barrier; F: facilitator
