# Supplementary material for: Developing a strategic understanding of telehealth service adoption for COPD care management: A causal loop analysis of healthcare professionals
Source: PLoS One. 2020 Mar 5;15(3):e0229619. doi: 10.1371/journal.pone.0229619 (PMC7058286; doi:10.1371/journal.pone.0229619)
Supplement: S2 Table — (DOCX) [file pone.0229619.s003.docx]

S2 Table. Main feedback loops considering different healthcare professionals

| HCP | Loop Nr. | Description | Loop Type |
| --- | --- | --- | --- |
| N | 1 | Adoption 🡪 *HPU* 🡪 ANDM 🡪 Adoption | R |
| N | 2 | Adoption 🡪 Adoption (Pa) 🡪 Enabled SM 🡪 Engagement in SM 🡪 Access to care 🡪 Workload 🡪 Change management 🡪 Adoption | R |
| N | 3 | Adoption 🡪 Adoption (Pa) 🡪 Enabled SM 🡪 Engagement in SM 🡪 Access to care 🡪 Workload 🡪 ANDM 🡪 Adoption | R |
| N | 4 | Adoption 🡪 Adoption (Pa) 🡪 Disease awareness🡪 Exacerbation recognition 🡪 Access to care  🡪 Workload 🡪 Change management 🡪 Adoption | R |
| N | 5 | Adoption 🡪 Adoption (Pa) 🡪 Disease awareness 🡪 Exacerbation recognition 🡪 Access to care🡪 Workload 🡪 ANDM 🡪 Adoption | R |
| N | 6 | Adoption 🡪 Adoption (Pa) 🡪 Enabled SM🡪 Engagement in SM 🡪 Access to care 🡪 Workload 🡪 Change management 🡪 ANDM 🡪 Adoption | R |
| N | 7 | Adoption 🡪 Adoption (Pa) 🡪 Disease awareness 🡪 Exacerbation recognition🡪 Access to care 🡪 Workload 🡪 Change management 🡪 ANDM 🡪 Adoption | R |
| N | 8 | Adoption 🡪 *HPU* 🡪 Patient risk 🡪 Perceived Value 🡪 Perceived Value 🡪 Adoption (Pa) 🡪 Enabled SM 🡪 Engagement in SM (including PA) 🡪 Access to care 🡪 Workload 🡪 Change management 🡪 Adoption | R |
| N | 9 | Adoption 🡪 *HPU* 🡪 Patient risk 🡪 Perceived Value 🡪 Perceived Value 🡪 Adoption (Pa) 🡪 Enabled SM 🡪 Engagement in SM 🡪 Access to care 🡪 Workload 🡪 ANDM 🡪 Adoption | R |
| N | 10 | Adoption 🡪 *HPU* 🡪 Patient risk 🡪 Perceived Value 🡪 Perceived Value (Pa) 🡪 Adoption (Pa) 🡪 Disease awareness 🡪 Exacerbation recognition 🡪 Access to care 🡪 Workload 🡪 Change management 🡪 Adoption | R |
| N | 11 | Adoption 🡪 *HPU* 🡪 Patient risk 🡪 Perceived Value 🡪 Perceived Value (Pa) 🡪 Adoption (Pa) 🡪 Disease awareness 🡪 Exacerbation recognition 🡪 Access to care 🡪 Workload 🡪 ANDM 🡪 Adoption | R |
| N | 12 | Adoption 🡪 *HPU* 🡪 Patient risk 🡪 Perceived Value 🡪 Perceived Value (Pa) 🡪 Adoption (Pa) 🡪 Disease awareness 🡪 Exacerbation recognition 🡪 Access to care 🡪 Workload 🡪 Change management 🡪 ANDM 🡪 Adoption | R |
| N | 13 | Adoption 🡪 *HPU* 🡪 Patient risk 🡪 Perceived Value 🡪 Perceived Value (Pa) 🡪 Adoption (Pa) 🡪 Enabled SM 🡪 Engagement in SM 🡪 Access to care 🡪 Workload 🡪 Change management 🡪 ANDM 🡪 Adoption | R |
| Ph | 1 | Adoption 🡪 Positive User experience 🡪 Perceived Value 🡪 Adoption | R |
| Ph | 2 | Adoption 🡪 Exacerbations monitoring 🡪 Physical activity management 🡪 Perceived Value 🡪 Adoption | R |
| Ph | 3 | Adoption 🡪 Positive User experience 🡪 Adoption motivation (Pa) 🡪 *HPU* 🡪 Adoption | R |
| Ph | 4 | Adoption🡪 Positive User experience 🡪 Adoption motivation (Pa) 🡪 *HPU* 🡪 Physical activity management 🡪 Perceived Value 🡪 Adoption | R |
| Ph | 5 | Adoption 🡪 Exacerbations monitoring 🡪 Physical activity management 🡪 Perceived Value 🡪 Adoption | R |
| Ph | 6 | Adoption 🡪 Positive User experience 🡪 Adoption motivation (Pa) 🡪 HPU 🡪 Physical activity management 🡪 Perceived Value 🡪 Adoption | R |
| Dr | 1 | Adoption 🡪 Perceived value 🡪 Adoption | R |
| Dr | 2 | Adoption 🡪 Workload 🡪 Adoption | B |
| Dr | 3 | Adoption 🡪 Workload 🡪 Perceived value 🡪 Adoption | R |
| Dr | 4 | Adoption 🡪 Workload 🡪 Change Management 🡪 Sustainability 🡪 Adoption | R |
| Dr | 5 | Adoption 🡪 Workload 🡪 Change Management 🡪 *HPU* 🡪 Perceived value 🡪 Adoption | R |
| Dr | 6 | Adoption 🡪 Perceived value 🡪 Adoption (Pa) 🡪Perceived Value (Pa) 🡪 Sustainability 🡪 Adoption | R |
| Dr | 7 | Adoption 🡪 Perceived value 🡪Champion presence 🡪 Selective activation of staff 🡪 Sustainability 🡪 Adoption | R |
| Dr | 8 | Adoption 🡪 Workload🡪 Perceived value 🡪 Adoption (Pa) 🡪 Perceived Value (Pa) 🡪 Sustainability 🡪 Adoption | R |
| Dr | 9 | Adoption 🡪  Workload 🡪 Change Management 🡪 *HPU* 🡪 Risk to patient 🡪 Perceived value 🡪 Adoption | R |
| Dr | 10 | Adoption 🡪 Workload 🡪 Change Management 🡪 Centralization of services 🡪 Relationship: patient-clinician 🡪 Perceived value 🡪 Adoption | R |
| Dr | 11 | Adoption 🡪 Workload 🡪 Perceived value 🡪 Champion presence 🡪 Selective activation of staff 🡪 Sustainability 🡪 Adoption | R |
| Dr | 12 | Adoption 🡪 Workload 🡪 Change Management 🡪 Centralization of services 🡪 Perceived Value (Pa) 🡪 Sustainability 🡪 Adoption | R |
| Dr | 13 | Adoption 🡪 Workload 🡪 Change Management 🡪 *HPU* 🡪 Centralization of services 🡪 Perceived Value (Pa) 🡪 Sustainability 🡪 Adoption | R |
| Dr | 14 | Adoption 🡪 Workload 🡪 Change Management 🡪 *HPU* 🡪 Centralization of services 🡪 Relationship: patient-clinician 🡪 Perceived value 🡪 Adoption | R |
| Dr | 15 | Adoption 🡪 Workload 🡪 Change Management 🡪 *HPU* 🡪 Perceived value 🡪 Champion presence 🡪 Selective activation of staff 🡪 Sustainability 🡪 Adoption | R |
| Dr | 16 | Adoption 🡪 Workload 🡪 Change Management 🡪 *HPU* 🡪 Perceived value 🡪 Adoption (Pa) 🡪 Perceived Value (Pa) 🡪 Sustainability 🡪 Adoption | R |
| Dr | 17 | Adoption 🡪 Workload 🡪 Change Management 🡪 *HPU* 🡪 Risk to patient 🡪 Perceived value 🡪 Champion presence 🡪 Selective activation of staff 🡪 Sustainability 🡪Adoption | R |
| Dr | 18 | Adoption 🡪 Workload 🡪 Change Management 🡪 *HPU* 🡪 Risk to patient 🡪 Perceived value 🡪 Adoption (Pa) 🡪 Perceived Value (Pa) 🡪 Sustainability -🡪 Adoption | R |
| Dr | 19 | Adoption 🡪 Workload 🡪 Change Management 🡪 Centralization of services 🡪 Relationship: patient-clinician 🡪 Perceived value 🡪 Adoption (Pa) 🡪 Perceived Value (Pa) 🡪 Sustainability 🡪 Adoption | R |
| Dr | 20 | Adoption 🡪 Workload 🡪 Change Management 🡪 Centralization of services 🡪  Relationship: patient-clinician-🡪 Perceived value 🡪 Champion presence 🡪 Selective activation of staff 🡪 Sustainability 🡪 Adoption | R |
| Dr | 21 | Adoption 🡪 Workload 🡪 Change Management 🡪 *HPU* 🡪 Centralization of services 🡪 Relationship: patient-clinician 🡪 Perceived value 🡪 Adoption (Pa) 🡪 Perceived Value (Pa) 🡪 Sustainability 🡪 Adoption | R |
| Dr | 22 | Adoption 🡪 Workload 🡪 Change Management 🡪 *HPU* 🡪 Centralization of services 🡪 Relationship: patient-clinician 🡪 Perceived value 🡪 Champion presence 🡪 Selective activation of staff 🡪 Sustainability 🡪 Adoption | R |

N = Nurse; Ph = Physiotherapist, Dr = Doctor; Pa = Patient; PA = physical activity; SM = self-management; HPU=holistic patient understanding; ANDM = autonomous nurse decision making; R = reinforcing feedback loop; B = balancing feedback loop.
