# Supplementary material for: Developing a strategic understanding of telehealth service adoption for COPD care management: A causal loop analysis of healthcare professionals
Source: PLoS One. 2020 Mar 5;15(3):e0229619. doi: 10.1371/journal.pone.0229619 (PMC7058286; doi:10.1371/journal.pone.0229619)
Supplement: S3 Table — (DOCX) [file pone.0229619.s004.docx]

**S4. Validation process.** Cause and effect variables for three HCPs validation

**Physiotherapists**

| Cause variable | Effect variable and polarity | Consultant 1 | Consultant 2 | Final decision for CLD |
| --- | --- | --- | --- | --- |
| *Perceived Value* | Adoption (+) | X | X | Include |
|  | Sustainability (+) | X | X | Include |
| *Perceived Value (Patient* | Adoption (+) | X | X | Include |
| *Centralization of service* | Relationships with Patient (-) | N | NS | Exclude |
|  | Holistic Patient understanding  (+/-) | X | X | Include |
|  | Perceived value (?) | X; NS | X; Add - | Include, Add - |
| *Perceived Age/Disease status(severity/activity)* | IT literacy (-) | X | X | Include |
|  | Adoption (?) | X; Add - | X | Include, Add - |
|  | Support for TH usage (+) | No | X | Exclude |
| *Perceived IT literacy* | Support for TH usage (-) | X | X | Include |
|  | Adoption (Patient) (+) | X | X | Include |
| Adoption (Patient) | IT literacy (+) (?) | NS | No | Exclude |
|  | Self-management | NS | No | Exclude |
|  | Holistic Patient understanding (+) | X | X | Include |
|  | Positive User experience (+) | X, add delay | X | Include, delay |
|  | Sustainability, dashed (+) | X | X | Include |
|  | Risk to Patient (+) | No | X | Exclude |
| Adoption | Positive User experience (+) | X, delay | X, delay | Include, delay |
|  | Exacerbation recognition dashed (+) | X | NS | Include |
|  | Access to care | No, if special physiotherapist | No | Exclude |
| Support for TH usage | Sustainability (+) | X | X | Include |
|  | Adoption (+) | X | X | Include |
| *Disease acceptance by Patient* | Self- management (+) | X | NS | Include |
| *Workload* | Adoption (-) |  |  |  |
| Usability (or design implications) | Adoption (+) | X | X | Include |
|  | Self –management motivation (+) | X | X | Include |
|  | Value perceived (+) | X | X | Include |
|  | Adoption (Patient) (+) | X | X | Include |
| Holistic Patient understanding | Adoption (+) | X | NS | Include |
|  | Physical activity management (+) | X | X | Include |
|  | Perceived value (Patient) (+) | No | No | Exclude |
|  | *Sustainability (dashed) (+)* | X | NS | Include |
| Positive User experience | Adoption (Patient) (+) | X | X | Include |
| Patient risk | *Perceived Value (-)* | *X* | *X, with a service, not a device* | *Include* |
| Relationship with Patient | Holistic Patient understanding (+) | X | X | Include |
|  | *Perceived Value (+)* | No | No | Exclude |
|  | *Perceived Value (Patient) (+)* | X | X | Include |
| Financial structures | Sustainability (+) | X | X | Include |
| Sustainability | Financial structures (+) | X | NS | Include |
| Self-management motivation | Adoption (Patient)(?) | NS | No | Exclude |
|  | Value perceived (+) | No | No | Exclude |
| Self-management training | Self-management (+) | X | X | Include |
|  | Workload dashed (+) | No, unless for the specific training | No | Exclude |
| Exacerbations monitoring | Physical activity (dashed) (+) | X | X | Include |
| Physical activity management | Exacerbations (dashed) (+) | X | X | Include |
|  | *Perceived Value (+)* | X | X | Include |
|  | *Perceived Value (Patient) (+)* | X | X | Include |
| Access to care | Adoption (Patient) (+) | X | X | Include |
| Knowledge gap (efficacy and cost-effectiveness) | *Perceived Value (-)* | X | X | *Include* |
|  | *Sustainability (-)* | NS | X | *Include* |
| Design implications | *Workload (-)* | *X* | *X* | *Include* |
| *Other proposals during the conversation* | | | | |
| Change management (CM) | *Perceived Value (Patient) (+) (delay)* |  | Patient of CM: Improved level of anxiety and depression | *Include as CM* |
| Change management | *Adoption (+)* | Multidisciplinary team | Patient of CM: Multidisciplinary team | *Include as C* |
| Change management | *IT literacy (+)* |  | Patient of CM: Relatives engagement | *Include as CM* |
| Change management | Adoption (Patient) (+) |  | Patient of CM:  Peer-to peer support | *Include as CM* |
| Financial structures | Adoption (+) |  | X | *Include* |
| Positive User experience | Value perceived (+) | X | X | *Include* |
| Positive User experience | Adoption (+) |  | X | *Include* |

Legend: + = positive polarity; - = negative polarity; ? = unknown polarity; red = authors hypothesis; NS = not sure; X = yes

**Physicians**

| Cause variable | Effect variable and polarity | Consultant 1 | Consultant 2 | Final decision for CLD | |
| --- | --- | --- | --- | --- | --- |
| Sustainability | Reimbursement for TH (+) | X | X | *Include* | |
|  | Adoption (+) | X | NS, delay | *Include, delay* | |
| Adoption | Workload (+) | X | X, it increases due to the false alarms | *Include* | |
|  | Value perceived (Dr) (+) | X | X | Include | |
|  | Nurse workload (+) | X | No | Exclude | |
| Adoption (Patient) | Value perceived (+) | X | X | Include | |
| *Value perceived (Patient)* | Sustainability (+) | X | X | Include | |
| *Value perceived (Dr)* | Adoption (Patient) (+) | X | X | Include | |
|  | Sustainability (+) | No | X | Exclude | |
|  | *Champion presence (+)* | X | X, delay | Include. delay | |
| Champion presence | Selective staff activation (+) | X | X | Include | |
|  | *Perceived value (+)* | X | X | Include | |
| Selective staff activation | Sustainability (+) | X | X, if service is provided by one nurse, then not | Include | |
| *Change Management* | Sustainability (+) | X | X | Include | |
|  | Workload (Dr) (-) | X | X | Include | |
|  | Decentralization of TH service (+) | NS | S | Include | |
|  | Nurse’s workload perception (delay)(+) |  | NS | Include | |
|  | *Workload (-)* | X | X | Include | |
| *Decentralization of service* | Perceived value (Patient) (-) | NS | X | Include | |
|  | Relationship between clinicians (-) | X | X | Include | |
|  | Workload (+) | X | No | Exclude | |
|  | *Perceived value (-)* | X | X | Include | |
| *Workload (Nurses)* | Workload (+) | Not always clear cut | Not always, example of independent nurses | Include | |
| *Workload* | Relationship (-) | No | NS | Exclude | |
|  | Change management (+) | X | NS | Include | |
|  | Adoption (-) | X | X | Include | |
| *Decision Making* | Algorithm triage interpretation (+) | X | X | Include | |
|  | Adoption (+) | X | X | Include | |
|  | Patient risk (-) | X | X | Include | |
|  | Change management (+) | X | X | Include | |
| *Usability* | Adoption (+) | X | X | Include | |
|  | Adoption (Patient) (+) | X | X | Include | |
|  | Activated self-management (+) | X | X | Include | |
|  | Perceived Value (Patient) (+) | X | X | Include | |
|  | Access to care (+) | X | X | Include | |
|  | Perceived value (+/-) | X | X | Include | |
| *Patient risks* | Perceived Value (-) | X | X | Include | |
| *Reimbursement for TH* | Sustainability (+) | X | X | Include | |
| *Relationship Patient-clinician* | Perceived Value (+) | X | X | Include | |
| *Activated Self-Management* | Exacerbation recognition (+) | X | 40% | Include | |
|  | Perceived value (+/-) | X | X | Include | |
| *Self-management training for Patient* | Self-management activation (+) | X | X | Include | |
| *Exacerbation recognition* | Perceived Value (+) | X | X | Include | |
| *Health Services* | Perceived value (+) | Access to care, is appropriate, then yes | Must be appropriate, not for nothing | Include | |
| *Use of triage algorithm* | “Right “algorithm cut-off value selection (+) (delay) | NS | Normally they are risk averse | Include | |
|  | Workload (+) | X | X | Include | |
|  | Workload (+) | X | X | Include | |
|  | Perceived value (+) | X | No | Exclude | |
|  | Usability (+) | X | X | Include | |
| “Right “algorithm cut-off value selection | Patient risk (AB) (-) | X | X | Include | |
| Holistic Patient understanding | Change management (+) | No | Opposite, it changes change management, move it | Include | |
|  | Risk to Patient (-) | X | X | Include | |
|  | Decentralization of service (+) | X | X | Include | |
|  | Perceived value (+) | Definitely; RCT is way to go; Before –after trials are always positive, not very interesting; If it is difficult, it doesn’t mean we don’t need to do it. I think the people who have had a negative trial advocate not for RCT | X | Include | |
| Knowledge gap | Perceived value (-) | X | X | | Include |
|  | Service usability (-) | X | X | Include | |
|  | Sustainability (-) | X | X | Include | |
| Disease status (AECOPD /obstruction severity based on clinical criteria) | Adoption (-) | X | X | Include | |
|  | Self-management activation (-/+) | X | X | Include | |
| *Other proposals during the conversation* | | | | | |
|  |  | Call sustainability routinization in your final CLD |  | Include | |
| *Decentralization of service* | Relationship between Dr – Patient (-) | X |  | Include | |
| *Change Management* | Holistic patient understanding (+) |  | X | Include | |

Legend: + = positive polarity; - = negative polarity; ? = unknown polarity; red = authors hypothesis; NS = not sure; X = yes

**Nurses**

| Factor | Effects on other factors | Consultant 1 | Consultant 2 | Final decision for CLD |
| --- | --- | --- | --- | --- |
| Perceived Value (Nurse) | Adoption (Patient) (+) | NS | X | Include |
|  | Sustainability (+) | X | NS | Include |
|  | TH training to nurses (+) | No | No | Exclude |
|  | Perceived Value (Patient) (+) | Not with time | X | Include |
| Perceived Value (Patient) | Adoption (Patient) (+) | X | X | Include |
|  | Sustainability (+) | NS | Motivation should be rewarded positive feedback; | Exclude |
| Right service initiation timing | Adoption (Patient) (+) | X | X | Include |
|  | Relationship N-P (+) | No | No | Exclude |
|  | Risk to the Patient (-) | NS | No | Exclude |
| Patient relatives’ involvement | Adoption (Patient) (+) | X | X | Include |
| Wiliness to include the Patient | Adoption (Patient) (+) | X | X | Include |
| Change management | Perceived Value (+) | No | Just leave change management; explain by the text; support by the team and specialist, confident in their skills | Exclude |
|  | Job satisfaction (+) | X | X | Include |
|  | Nurse decision making (+)(delay) | X | X | Include |
|  | Adoption (+) | X | X | Include |
|  | Workload (-)(delay) | X | X | Include |
| Presence of TH technology support | Workload (-) | X | Dependence of size of organization | Include |
|  | Equipment issues (-) | X | X | Include |
|  | Nurse confidence in the system (+) | X, Nurse needs to take responsibility. We can solve ourselves. Pollute tech, support. Forget to change batteries. | X | Include |
|  | Sustainability (+) | X | X | Include |
| Disease status | Adoption (Patient)(+) | No | No | Exclude |
|  | Right service initiation timing (-) | X | X | Include |
| IT literacy (Patient) | Willingness to include the Patient (+) | X | X | Include |
|  | Perceived Value (+) | No, we have 1 h meeting as standard | NS | No |
|  | Adoption (Patient)(+) | X | X | Include |
| Champion presence | Adoption (+) | X | X | Include |
|  | Selective staff activation (+) | X | X | Include |
|  | Change management (+) | X | X | Include |
|  | Sustainability (+) | X | NS | Include |
| Selective staff activation (+) | Adoption (+) | X | X | Include |
| Adoption motivation (Patient) | Workload (+) | X | X | Include |
|  | Sustainability (+) | X | X | Include |
|  | Physical activity management (+) | X | NS | Include |
|  | Disease awareness (+) | X | X | Include |
| Data accessibility (Nurse) | Comorbidities detection (+) | X | X | Include |
|  | Nurse decision making (+) | NS | X | Include |
| Adoption (Nurse) | Relationship N (O/S) | NS | No, Professional rules; depends on the intervention; trained to do more  decision themselves; remove this | Exclude |
|  | Adoption (Patient)(+) | X | X | Include |
|  | Nurse professional security (-/+) | NS | X | Include |
|  | Holistic understanding (+) | X | X | Include |
|  | Workload (Nurse)(+) | X | No | Exclude |
| Positive user experience | Nurse confidence in the system (+) | X | X | Include |
|  | Adoption motivation (Patient)(+) | X | X | Include |
| Nurse decision making (doctor involvement, triage decision making; records update) | Adoption (+) | X | X | Include |
|  | Job satisfaction (+) | X | X | Include |
|  | Adoption (-/+) | X | X | Include |
| Lack of TH standards | Nurse decision making (+) | X | X | Include |
| Job seniority | Nurse decision making (+/-) | X | X | Include |
|  | Job satisfaction (+) | X | X | Include |
| Equipment issues (logistics and not correct manuals) | Adoption (Patient)(-) | No | NS | Exclude |
|  | Value perceived (Patient)(-) | NS | No | Exclude |
|  | value perceived (-) | No | X | Exclude |
|  | Nurse decision making (+) | X | X | Include |
|  | Confidence in the system (-) | X | X | Include |
|  | Workload (+) | X | X | Include |
|  | User experience (-) | X | X | Include |
|  | Adoption (-) | No | NS | Exclude |
| Workload (Nurse) | Right service initiation timing (-) | X | X | Include |
|  | Job satisfaction (-) | X | X | Include |
|  | Change management (+) | X | X | Include |
| Use of triage system | Workload (+) | NS | X | Include |
|  | Enabled SM(+) | X | X | Include |
|  | Risk to the Patient (+)(it goes via triage) | X | No | Exclude |
| Disease awareness | Exacerbation recognition (+) | X | X | Include |
| Usability (TH implications) | Knowledge gap (-) | X | X | Include |
| Holistic Patient understanding | Nurse confidence in the system (+) | X | X | Include |
|  | Patient risk (AB/” shy” Patients)(-) | X | X | Include |
|  | Nurse decision making (+) | X | X | Include |
|  | Use of triage system (+) | X | X | Include |
|  | Value perceived (+) | No | X | Exclude |
| Patient risk (AB/”shy” Patients) | Value perceived (-) | X | X | Include |
| Relationship N-P | Adoption (Nurse) (+) | No | No | Exclude |
|  | Job satisfaction (+) | X | X | Include |
|  | Holistic Patient understanding (+) | X | X | Include |
| Engagement in SM | Perceived value (+) | X | X | Include |
|  | Perceived Value (P)(+) | No | No | Exclude |
|  | Disease awareness (+) | X | X | Include |
|  | Access to care(-)(delay) | X | X, not sure about polarity | Include |
| Enabled SM | Engagement in SM (+)(delay) | X | X | Include |
| Exacerbation recognition | Access to care (+) | X | X | Include |
| Access to care | Value perceived (Nurse) (+) | X | X | Include |
|  | Nurse decision making (+) | X | No | Exclude |
|  | Value perceived (+) | X | X | Include |
|  | Relationship N-D (+) | Ns | No | Exclude |
|  | Workload (+) | X | X | Include |
| Knowledge gap (efficacy and cost-effectiveness) | Sustainability (-) | X | X | Include |
| *Other proposals during the conversation* | | | | |
| Change management (CM) | Sustainability (+) | CM: communication plan |  | CM include |
| Change management (CM) | Adoption (+) | CM: communication plan |  | CM include |
| Change management (CM) | Right service initiation timing (+) |  | X | Include |
| Adoption motivation (Patient) | Change management |  | CM:TH training to Patient by N (+) | CM include |
| Workload (Nurse) | Nurse decision making (+) |  | X | Include |
| Adoption motivation (Patient) | Enabled SM (+) |  | X | Include |

Legend: + = positive polarity; - = negative polarity; ? = unknown polarity; red = authors hypothesis; NS = not sure; X = yes
