# Supplementary material for: Developing a strategic understanding of telehealth service adoption for COPD care management: A causal loop analysis of healthcare professionals
Source: PLoS One. 2020 Mar 5;15(3):e0229619. doi: 10.1371/journal.pone.0229619 (PMC7058286; doi:10.1371/journal.pone.0229619)

S1. Complete CLD for three HCPs

Physicians perspective

Nurses perspective


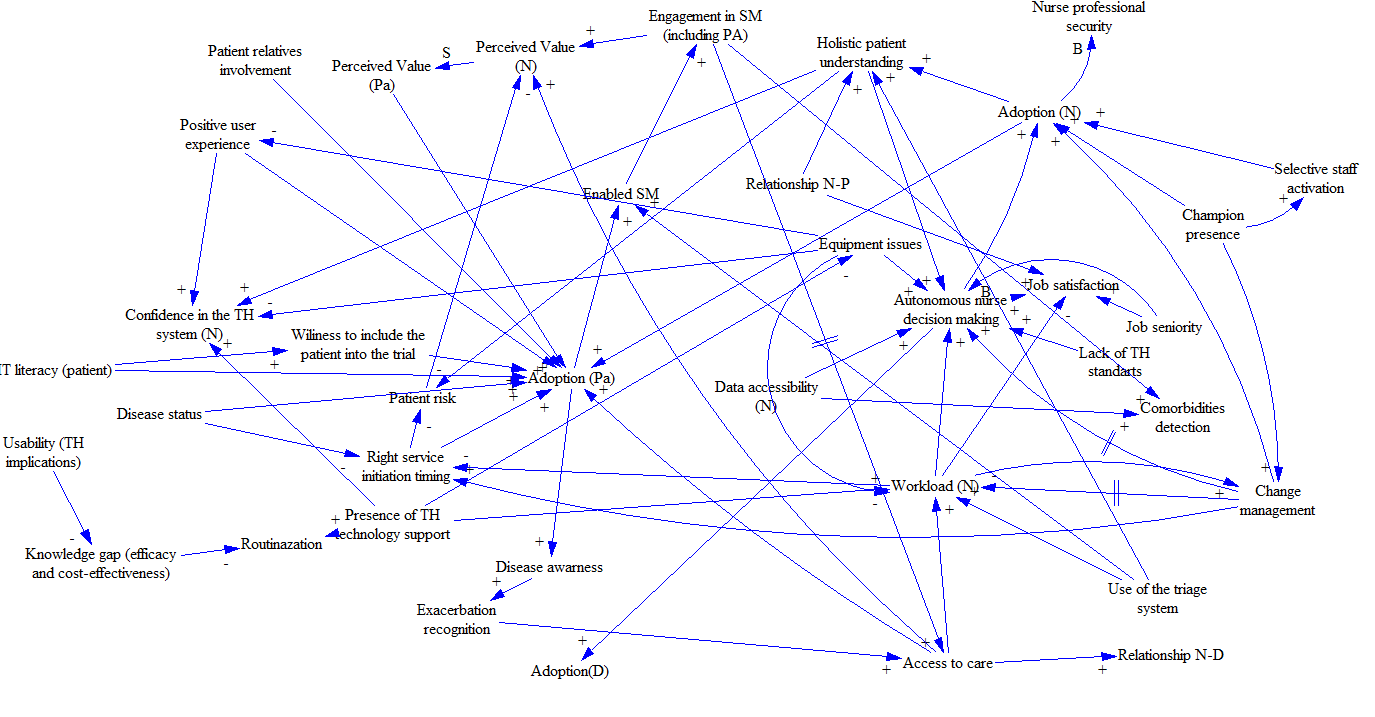


Physiotherapists perspective


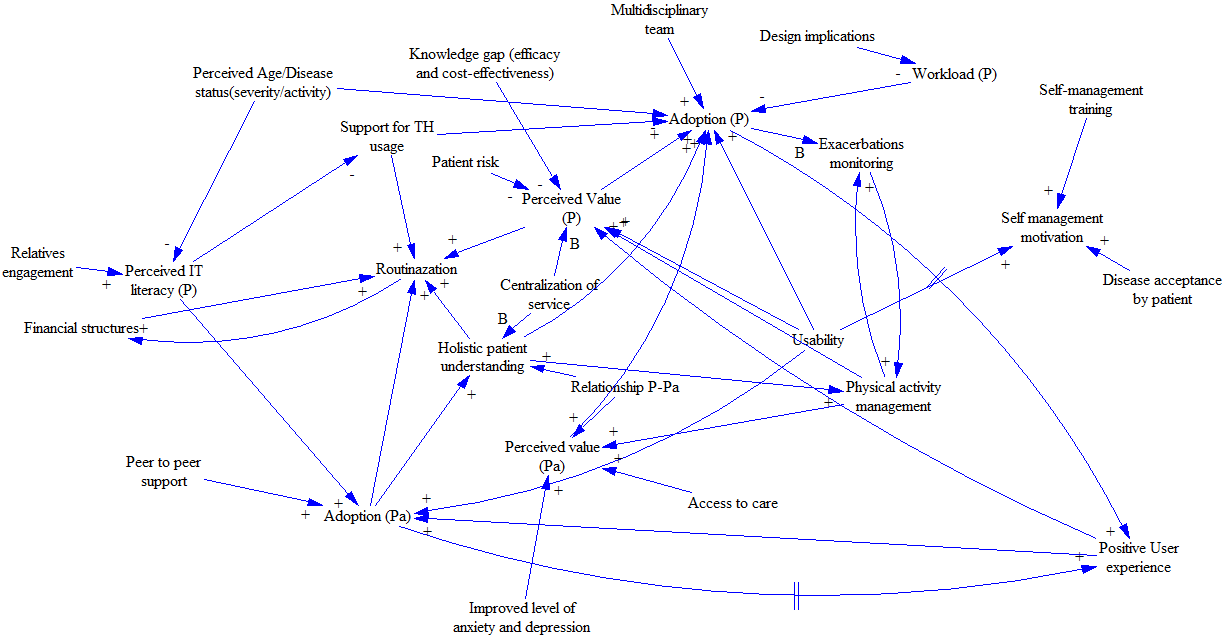

Supplement: S1 Fig — (DOCX) [file pone.0229619.s005.docx]
